# Growth factor signaling predicts therapy resistance mechanisms and defines neuroblastoma subtypes

**DOI:** 10.1038/s41388-021-02018-7

**Published:** 2021-09-23

**Authors:** Timofey Lebedev, Elmira Vagapova, Pavel Spirin, Petr Rubtsov, Olga Astashkova, Alesya Mikheeva, Maxim Sorokin, Uliana Vladimirova, Maria Suntsova, Dmitry Konovalov, Alexander Roumiantsev, Carol Stocking, Anton Buzdin, Vladimir Prassolov

**Affiliations:** 1 Department of Cancer Cell Biology, Engelhardt Institute of Molecular Biology, Russian Academy of Sciences, Moscow, Russia; 2 Center for Precision Genome Editing and Genetic Technologies for Biomedicine, Engelhardt Institute of Molecular Biology, Russian Academy of Sciences, Moscow, Russia; 3 Moscow Institute of Physics and Technology (National Research University), Moscow Region, Russia; 4 Group for Genomic Regulation of Cell Signaling Systems, Shemyakin-Ovchinnikov Institute of Bioorganic Chemistry, Moscow, Russia; 5 Department of Bioinformatics and Molecular Networks, OmicsWay Corporation, Walnut, CA USA; 6 Institute of Personalized Medicine, Sechenov First Moscow State Medical University, Moscow, Russia; 7 D. Rogachyov Federal Research Center of Pediatric Hematology, Oncology and Immunology, Moscow, Russia; 8 Research Department Cell and Gene Therapy, Department of Stem Cell Transplantation, University Medical Center Hamburg-Eppendorf (UKE), Hamburg, Germany; 9 Heinrich-Pette-Institute, Leibniz Institute for Experimental Virology, Hamburg, Germany

**Keywords:** Cancer genomics, Paediatric cancer

## Abstract

Neuroblastoma (NB) has a low frequency of recurrent mutations compared to other cancers, which hinders the development of targeted therapies and novel risk stratification strategies. Multikinase inhibitors have shown potential in treating high-risk NB, but their efficacy is likely impaired by the cancer cells’ ability to adapt to these drugs through the employment of alternative signaling pathways. Based on the expression of 48 growth factor-related genes in 1189 NB tumors, we have developed a model for NB patient survival prediction. This model discriminates between stage 4 NB tumors with favorable outcomes (>80% overall survival) and very poor outcomes (<10%) independently from MYCN-amplification status. Using signaling pathway analysis and gene set enrichment methods in 60 NB patients with known therapy response, we identified signaling pathways, including EPO, NGF, and HGF, upregulated in patients with no or partial response. In a therapeutic setting, we showed that among six selected growth factors, EPO, and NGF showed the most pronounced protective effects in vitro against several promising anti-NB multikinase inhibitors: imatinib, dasatinib, crizotinib, cabozantinib, and axitinib. Mechanistically kinase inhibitors potentiated NB cells to stronger ERK activation by EPO and NGF. The protective action of these growth factors strongly correlated with ERK activation and was ERK-dependent. ERK inhibitors combined with anticancer drugs, especially with dasatinib, showed a synergistic effect on NB cell death. Consideration of growth factor signaling activity benefits NB outcome prediction and tailoring therapy regimens to treat NB.

## Introduction

Neuroblastoma (NB) is the most common pediatric extracranial solid tumor and is responsible for ~8% of all childhood cancer cases [1]. One of NB hallmarks is high clinical heterogeneity: low-risk NB treated without chemotherapy and high-risk NB having a survival rate of <50% even after multimodal therapy [2]. Current NB risk stratification is mainly based on an assessment of clinical data, tumor histology, and genetic aberrations, such as MYCN-amplification (20% of all NB and 50% of high-risk NB) and 11q aberrations (20–45% of all NB) [3, 4]. NB has a very low frequency of recurrent mutations, and for the majority of high-risk NB driver mutations or genetic aberrations have not been yet identified [5].

Despite lack of mutations in growth factor receptors and other receptor tyrosine kinases (RTKs), except ALK (mutated in 9% of all NB), which has a strong correlation with MYCN-amplification [6], several studies showed that NBs are highly dependent on RTK signaling, such as KIT [7, 8], PDGFRB [8], MET [9], and RET [9, 10]. Several multikinase inhibitors such as imatinib, dasatinib, and crizotinib that target several RTKs, including KIT, PDGFRs, and ALK, have been tested in clinical trials for high-risk NB treatment (clinical trials: NCT02559778, NCT00030667, NCT01467986, NCT00788125, NCT00939770, and NCT03126916). Although high-risk NB showed an improved initial response to multikinase inhibitors, many tumors lose sensitivity to these drugs [11–15], suggesting that malignant cells’ adaptation to multikinase inhibitors mainly hindered such therapy’s effectiveness.

Several large-scale studies identified novel prognostic markers for high-risk NB based on rare somatic mutations and genetic alterations [5, 16], *TERT* gene rearrangements [17], and chromothripsis [18]. Considering the high dependency of NB cells on growth factor signaling, we chose a different approach. We investigated which signaling pathways, mainly growth factor-related, are associated with NB progression and therapy escape and how these pathways can be effectively targeted. We were also interested whether activation of certain growth factor signaling pathways would affect efficacy of kinase inhibitors.

## Results

### Growth factor-related genes expression defines NB subtypes

**Fig. 1:**
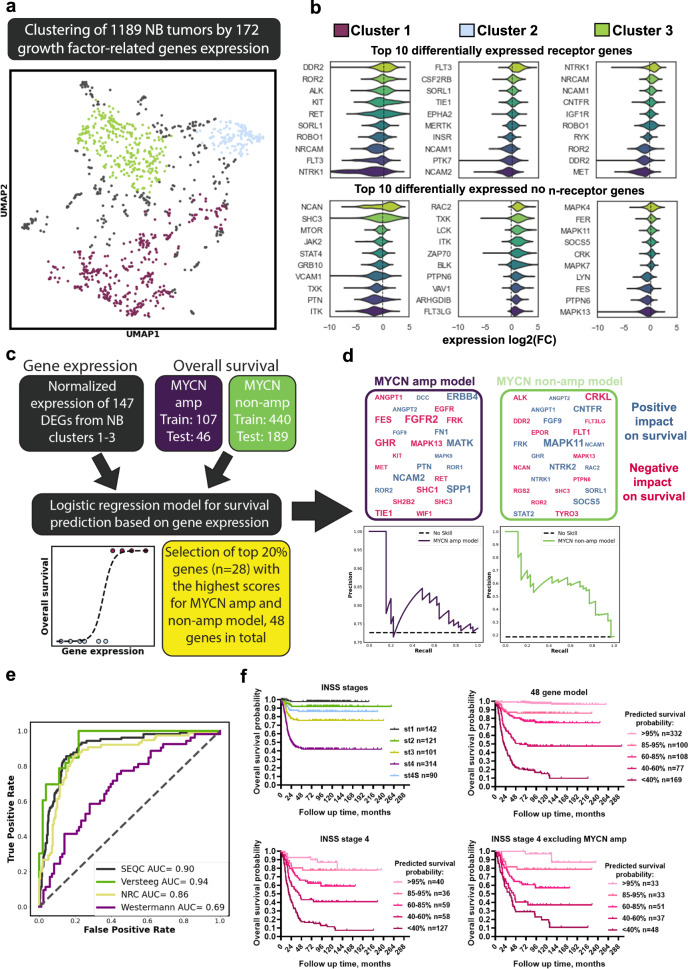
Growth factor-related genes expression in NB tumors. **a** NB tumors (*n* = 1189) clustering based on UMAP and HDBSCAN algorithms. NB tumor Kocak, NRC, Versteeg, Maris, and Westermann datasets were downloaded from the R2: Genomics analysis and visualization platform (Table S1). Additional UMAP plots are provided in Fig. S1, and UMAP data is provided in Table S2. “No cluster” indicates samples that did not belong to any cluster based on HDBSACN clustering. **b** Top ten differentially expressed receptor and non-receptor genes (DEGs) for each cluster with lowest *p* values after FDR correction from 168 genes used for UMAP. Gene expression is provided as log2 fold change compared to mean expression across all samples. DEGs for each cluster are provided in Table S2. **c** Scheme for creation of survival prediction model for patients with MYCN amplified (amp) and non-amplified (non-amp) NB tumors based on 147 cluster DEGs expression using logistic regression. **d** Genes used in the final model for MYCN amplified and non-amplified tumors and precision-recall curves for each part of the final model. Font size is proportional to the absolute weight coefficient of each gene in the model. **e** ROC curves for prediction of patient survival in individual datasets using the final model. AUC values are provided for each dataset. **f** Kaplan–Meier survival analysis of NB patient survival from Cangelosi dataset using either INSS staging or 48 growth factor-related gene prediction model. Survival analyses for INSS stage 4 tumors (including MYCN amplified) and stage 4 tumors excluding MYCN amplified are shown at the bottom.

First, we analyzed how diverse NB tumors are, based on growth factor-related genes expression. We selected 1189 NB tumors from five independent datasets with available gene expression and disease outcome data from R2: Genomics analysis and visualization platform: Kocak, NRC, Versteeg, Maris, and Westermann [18–23] (Table S1) and 277 genes (172 present in all datasets) encoding cell receptor ligands, including growth factors, their receptors, and downstream kinases from HUGO Gene Nomenclature Committee (HGNC) database (Table S2). Our analysis revealed three clusters (Figs. 1a, S1a), each characterized by the differentially expressed (DEGs) receptors or their downstream kinases (Fig. 1b, Table S2). Cluster 1 had the highest percentage of MYCN amplified (48%) (Fig. S1a, b) and was characterized by higher expression of several receptors such as *DDR2*, *ROR2, ALK*, and *RET* (Fig. 1a, Table S2). Cluster 3 had the lowest percentage of MYCN amplified tumors (1%) (Fig. S1a, b) and had a high expression of *NTRK1, NRCAM*, and *NCAM1* (Fig. 1b, Table S2). High expression of these receptors is associated with a better prognosis for NB patients [24, 25]. Cluster 2 had a higher expression of several receptors and kinases associated with immune cells: *FLT3, CSF2RB, RAC2*, *LCK*, *ITK*, and *ZAP70* (Fig. 1b, Table S2) [26], potentially indicating immune infiltration. As expected, patients from cluster 1 with a prevalence of MYCN amplified tumors had the worst prognosis, and patients in cluster 3 had a very favorable prognosis (Fig. S1c).

The expression of many receptors from clusters DEGs, including several potential drug targets, was significantly associated with NB patient survival (Fig. S2a, Table S3). Interestingly, we found that several receptors had a different association with survival prognosis, depending on whether MYCN amplified tumors were included in the analysis. *KIT*, *FGFR2*, and *ROR2* had a much more significant association with the worse outcome when MYCN amplified tumors were included in the analysis (Fig. S2b). *MET*, *RYK*, *DDR2*, *ALK*, and *RET* were strongly associated with poor outcomes independently from the inclusion of MYCN amplified tumors (Fig. S2b). All these receptors are higher expressed in cluster 1 (Table S2), supporting our observation that high expression of these receptors may mimic aggressive MYCN amplified phenotype, even in MYCN non-amplified tumors. Interestingly, higher *EPOR* expression, upregulated in cluster 1 tumors without MYCN-amplification (Table S2), was strongly associated with worse prognosis only in NB tumors without MYCN-amplification (Fig. S3).

To evaluate which growth factor-related genes expression could provide beneficial information for predicting patient survival, we performed logistic regression with elastic net regularization to train a model for NB outcome prediction based on gene expression. We selected 147 genes that were differentially expressed in MYCN amplified or MYCN non-amplified tumors from different clusters (Fig. 1c). We used an integrated and batch-controlled dataset with available gene expression and survival data [27], which contains data for large amount of NB samples (*n* = 786). To generate a model which can be applied and tested on independent datasets, we normalized gene expression data by dividing each gene expression by its mean expression in a whole dataset. We separately trained survival prediction models for MYCN amplified and MYCN non-amplified tumors since many genes have a different impact on survival prognosis for these tumor types.

After the first round for each model, we selected 20% genes (*n* = 28) with the highest absolute weight coefficients (Fig. 1c). In total, we selected 48 genes, and only eight genes were common for MYCN amplified and non-amplified models (Fig. 1d). Then we used these genes to generate the final model, which separately predicts the survival of patients with or without MYCN-amplification (Table S4). Prediction accuracies for both cases were then checked on a test dataset using precision-recall (Fig. 1d). When our model was applied to other datasets, which were independently normalized, we achieved good ROC AUC values (0.69–0.94) for datasets that were partially included in our train/test dataset (Versteeg and SEQC), as well as for completely independent NRC and Westermann datasets (Fig. 1e). Next, we divided NB patients into five groups, based on survival predicted by our 48 gene model: >95%, 85–95%, 60–80%, 40–60%, and <40% survival probability. We show that our predicted survival probability matches actual survival probability and potentially provides better risk stratification than INSS staging (Fig. 1f). Moreover, our model allows distinguishing between INSS stage 4 patients with favorable and very poor survival prognosis, even when MYCN amplified highly aggressive tumors were excluded from analysis (Fig. 1f).

### Activated growth factor signaling is associated with metastasis, poor outcome, and relapse incidence in NB patients

**Fig. 2:**
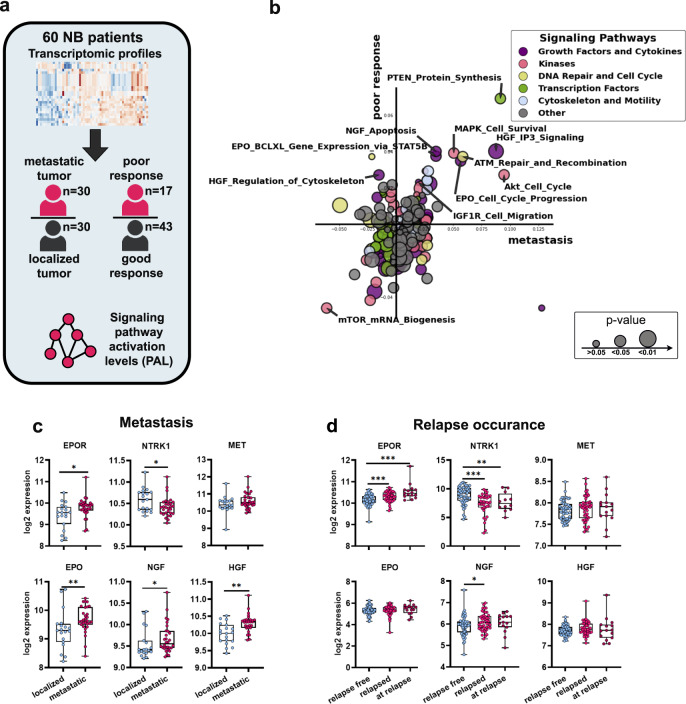
EPO, HGF, and NGF signaling pathways are upregulated in aggressive NB tumors. **a** Experimental design for signaling pathway analysis in 49 NB, 6 ganglioneuroma, and 5 ganglioneuroblastoma patients. **b** 2D plot showing association of each signaling pathway with metastasis and response to the therapy. Axes show pathway activation levels (PALs) in patients with poor therapy response vs. good response (poor response axis) and in metastatic vs. localized tumors (metastasis axis). Higher values indicate that certain pathway is strongly upregulated in either patients with poor therapy response or metastasis. Heatmap for top 100 changed pathways in metastatic and resistant tumors is provided in Fig. S4. **c** Expression of EPO, EPOR, NGF, NTRK1, HGF, and MET genes in localized (*n* = 19) and metastatic (*n* = 30) NB tumors. **d** Gene expression in primary NB tumors that did not relapse (relapse-free, *n* = 56), relapsed later (*n* = 46), and in samples obtained from already relapsed NB tumors (*n* = 15). Transcriptomic data for relapsed patients was taken from the Seeger NB dataset [30]. Median values, 25 to 75th percentiles, individual data points, minimal and maximal values are shown on box and whiskers graphs. *p value < 0.05; **<0.01; ***<0.001 as calculated by Mann–Whitney *U* test.

We investigated the association of signaling pathways activities with tumor metastasis and response to the therapy to expand our observations that growth factor signaling contributes to the development of aggressive NB phenotypes. To calculate association of signaling pathways with therapy response and metastasis we used gene expression profiles for 60 NB, ganglioneuroma, and ganglioneuroblastoma tumors (41 samples from previously published dataset and 19 new samples) [28] with extensive clinical data, including presence of metastasis, and response to the therapy, and applied the Oncobox algorithm [29] (Fig. 2a, Table S5, Fig. S4).

We calculated changes in signaling pathways for patients with metastatic vs. localized tumors and responder vs. nonresponder patients (Fig. 2a, Tables S5). Among signaling pathways with the highest association with metastatic NBs and NBs with poor response to the therapy were growth factor signaling pathways: erythropoietin (EPO), neural growth factor (NGF), and hepatocyte growth factor (HGF) (Fig. 2b, Table S5). MAPK-dependent cell survival was also associated with metastatic and poor response tumor phenotypes, and MAPK signaling is one of the main pathways activated by growth factors.

Expressions of both *EPOR* (*p* < 0.05) and *EPO* (*p* < 0.01) genes were higher in metastatic NB tumors, thus suggesting the existence of EPO/EPOR autocrine loop in aggressive NB tumors (Fig. 2c). In agreement with the previous findings that *NTRK1* expression (encodes NGF receptor TrkA) is associated with favorable prognosis [24], *NTRK1* was downregulated in metastatic tumors, although *NGF* expression was elevated. Next, we analyzed gene expression in primary and relapsed NB tumors without *MYCN* amplification from the Seeger dataset [30]. We found higher *EPOR* expression in 46 primary tumors of patients who later had a relapse than in 56 patients without relapse (*p* < 0.01), and *EPOR* expression was even higher in 15 tumors directly isolated from relapsed NB tumors *EPOR* (*p* < 0.001) (Fig. 2d).

Notably, higher *EPOR* expression was linked to a worse prognosis in patients without *MYCN* gene amplification in NB datasets (4/7 with *p* < 0.05) (Fig. S3). While *MET* expression (encodes HGF receptor) was not associated with metastasis or relapse, its higher expression strongly correlated with considerably worse outcomes (Fig. S2a, b). *HGF* expression was elevated in metastatic NB (Fig. 2c) but had no association with relapse events (Fig. 2d). Our findings suggest that EPO may contribute to relapses and overall NB progression, and HGF and NGF might contribute to metastases’ formation and tumor cell survival during therapy.

### Growth factor receptors are involved in similar cellular processes in NB

**Fig. 3:**
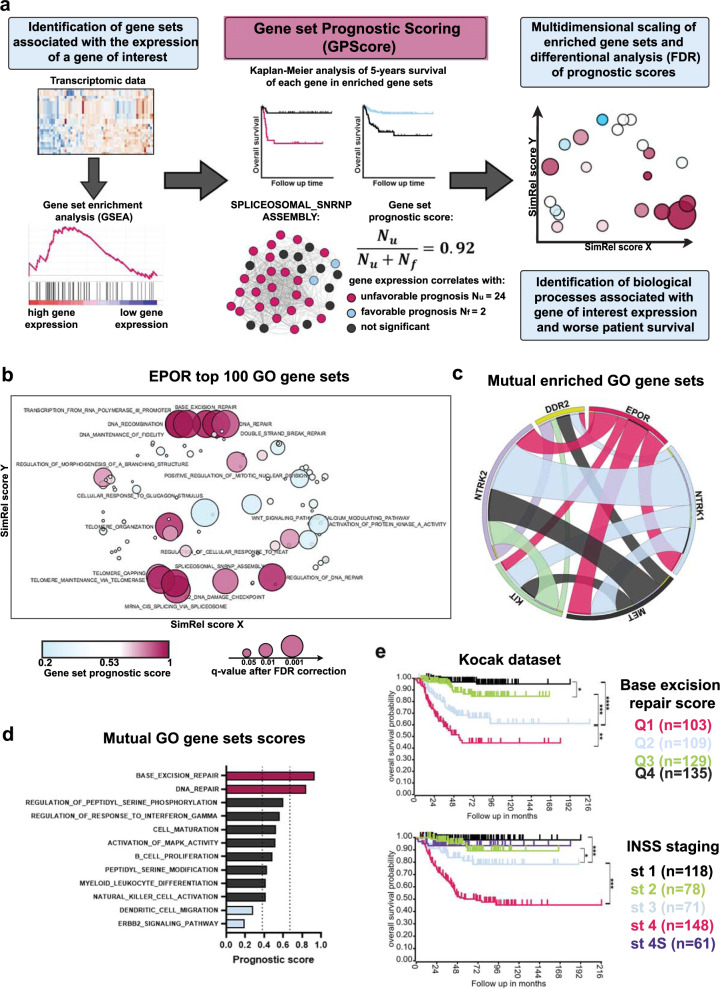
Gene set prognostic scoring for growth factor receptors in NB tumors. **a** Design of a GPScore (Gene set prognostic scoring) algorithm for calculation of gene prognostic scores, data visualization, and analysis. SPLICEOSOMAL_SNRNP_ASSEMBLY gene set is provided as an example. **b** Multidimensional scaling of enriched gene sets associated with EPOR expression in NB patients. Only gene sets with prognostic scores that passed the FDR test (*q* value < 0.01) are marked. **c** Circos plot showing the number of shared gene sets calculated by GSEA for each gene pair. The width of ribbons is proportional to the number of shared enriched gene sets. **d** Prognostic scores for 12 enriched gene sets shared by at least 5 of 6 genes. Dot lines indicate 3 sigma interval for prognostic scores distribution for 20 randomly generated gene sets. **e** Kaplan–Meier survival analysis for Kocak dataset (*n* = 476) based on base-excision repair score (divided into quartiles from highest Q1 to lowest Q4 scores) and INSS stages. A similar analysis for NRC and Versteeg datasets is provided in Fig. S5b.

To understand which biological processes are activated by growth factor receptors in NB tumors and contribute to aggressive tumors development, we developed a new method: **G**ene set **P**rognostic **S**coring (GPScore). This method is based on gene set enrichment analysis (GSEA) [31] combined with the calculation of prognostic scores for each gene set based on clinical data (Fig. 3a, Table S6). Although the association of individual gene expression with prognosis does not indicate its involvement in the development of tumor phenotype, we reasoned that if most genes involved in the same process are associated with poor prognosis, this process is more likely to be involved in aggressive phenotype development. Thus we calculated prognostic scores (Fig. 3a) based on the Kaplan–Meier survival analysis for each gene within each gene set, using overall survival data from three NB datasets covering 847 NB samples: Versteeg (*n* = 88) [18], NRC (*n* = 283) [22], and Kocak et al. (*n* = 476) [20] (Table S7). Prognostic score calculation showed high consistency for independent datasets of various sizes (Fig. S5a) and provided statistically significant results compared to randomly generated gene sets (Fig. S5b, and Methods section). We identified 25 gene sets for EPOR with prognostic score values different from randomized gene sets (FDR *q* < 0.01; Table S7). DNA repair, RNA stability, and splicing gene sets formed distinct clusters for *EPOR* (Fig. 3b) and had a high prognostic score, i.e., correlated with poor overall survival. These results suggest that *EPOR* may contribute to aggressive NB phenotype development through these biological processes.

In addition to *EPOR*, we selected several other receptor genes associated with poor outcomes (Fig. S2a, b) and a sufficient number of enriched GO gene sets (Table S5): *NTRK2*, *MET*, *KIT*, and *DDR2*. We also included *NTRK1* because increased NGF signaling was associated with metastatic and poor response NB (Fig. 2b, c). For each receptor, we performed GPScore analysis and compared enriched GO gene sets. We found that these receptor genes shared 12 gene sets (shared by at least 5 of 6 genes) (Fig. 3d). Among these gene sets, base-excision DNA repair showed the highest prognostic score, shared by all receptor genes except (Fig. 3d, Table S6, S7). We calculated base-excision repair scores for each NB tumor sample from three datasets (Versteeg, NRC, and Kocak) as a mean squared expression of all genes listed in base-excision repair GO, and divided patients into four groups (quartiles) based on the calculated score. Compared to INSS staging, our score provided better risk stratification, especially for intermediate-risk patients: Q4, Q3, and Q2 had better discrimination than stages 1, 2, 3, and 4S (Fig. 3e, S5c). Thus, we show that the GPScore method can help identify processes related to particular genes and potentially involved in aggressive tumors development and develop new risk stratification strategies based on gene expression patterns.

### Growth factors NGF and EPO protect NB cells from anticancer drugs

We identified increased growth factor signaling as a potential therapy-induced cell death escape mechanism in NB tumors. Since growth factor signaling is the main target for many kinase inhibitors, we were particularly interested in whether activation of alternative growth factor signaling pathways would affect kinase inhibitors efficacy. To test our hypothesis, we selected six drugs: vincristine, used for NB therapy, and five multikinase inhibitors and measured how the addition of growth factors affected the survival of six NB cell lines: SH-SY5Y, SK-N-BE, LAN-1, Kelly, SK-N-AS, and SK-N-SH. We analyzed mutation data for 46 NB cell lines from the CCLE database [32] to select potential kinase inhibitors and compared mutation frequency in cell lines with patient tumors from the TARGET dataset [5]. We found that many mutations in genes, such as *RP1L1*, *TP53*, *CACNA1H, VWF, PKD1L1*, and *TP53*, were highly overrepresented in NB cell lines, each mutation was present in more than 40% cell lines compared to NB patients, in which these mutations were found for less than 1% tumors (Fig. S6a, Table S8). Thus we selected 18 mutations that are present in at least 2% of NB patients and present in at least one cell line from our panel. The most common mutations were in *ALK* (4/6 cell lines), *TTN* (3/6), *RYR1*, and *MUC17* (2/6) genes (Table S1). Then we used published algorithm [33] to search the DSigDB database for FDA approved drugs which can directly target either 147 growth factor-related DEGs for NB clusters (Fig. 1a, Table S2) or 18 found mutations (Table S8). We selected the potential anti-NB drug crizotinib, which among its 36 targets, also inhibits mutant ALK isoform (Table S8). We also selected KIT/ABL inhibitors imatinib, dasatinib, tested in clinical trials for NB treatment, and two additional drugs with different main targets axitinib (VEGFRs, FGFRs), and cabozantinib (MET, RET, AXL). A full list of drug targets is provided in Table S8.

**Fig. 4:**
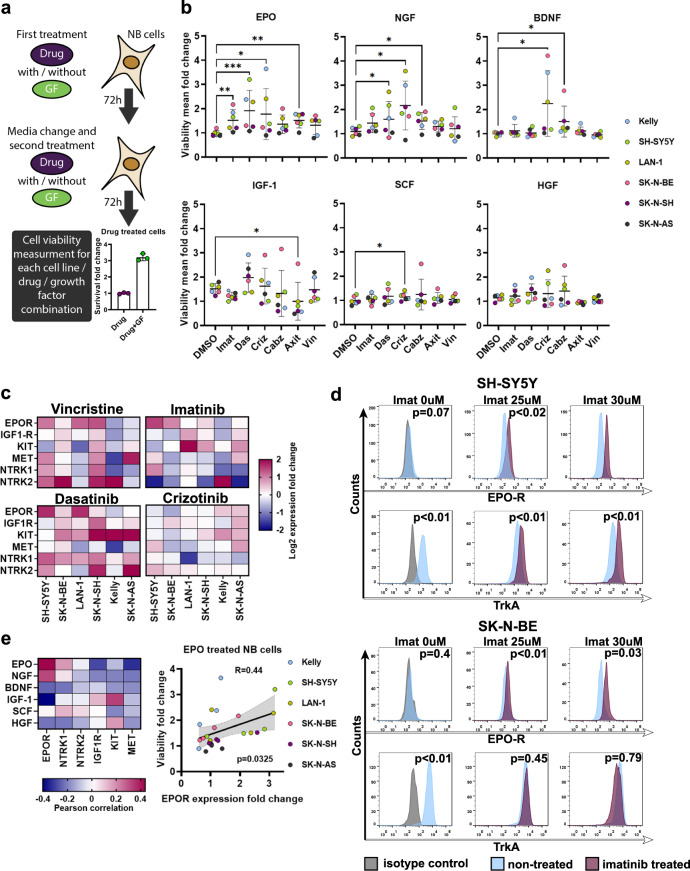
EPO and NGF protect NB cells from anticancer drugs. **a** Experiment design for studying growth factor-induced cell survival. NB cells were treated for 72 h with drugs with/without growth factors (GF) for 72 h, then growth media was changed, new drugs and GFs added in the same concentrations, and cell viability measured after another 72 h. Drug action on cell survival in absence of GF is shown in Fig. S7a, drug concentrations are provided in Table S9. **b** Growth factor-induced cell survival for NB cells treated with anticancer drugs. Each dot represents mean viability fold change for a particular cell line treated with the drug in combination with 100 ng/ml GF compared to cells treated with the drug alone. Each experiment for each cell line and drug/GF combination was performed in triplicates. Mean changes in cell viability for all six NB cell lines treated with drug/GF combination were compared with cells treated with DMSO and GF. Mean and SD values are shown, Friedman test was used to determine statistically significant differences. *p value < 0.05; **<0.01; ***<0.001; ****<0.0001. **c** Heatmaps of gene expression fold changes in NB cells treated with imatinib, dasatinib, crizotinib, and vincristine compared to cells treated with DMSO (mock treatment). Expression changes were measured by real-time PCR. **d** Staining with anti-EPOR and anti-TrkA antibodies of SH-SY5Y and SK-N-BE cells after imatinib treatment for 72 h and measured by flow cytometry. Each experiment was performed in triplicate, the most representative histograms are shown, *p* values are calculated by a two-sided *t* test. **e** Pearson correlations of GF-induced protective effect and receptors expression change in drug-treated NB cells.

To test how growth factors affect NB cell survival, we selected six growth factors: EPO, NGF, HGF, and IGF-1 as they had the most upregulated pathways in tumors with poor response to the therapy (Fig. 2b). Additionally we selected SCF, and BDNF, as their receptors KIT and NTRK2 high expression is associated with poor NB survival (Fig. S2a), and these growth factor has reported ability to protect NB cells from anticancer drugs [34, 35]. NB cells were treated with drugs with and without the addition of growth factors for 6 days (Fig. 4a). Drug concentrations which reduce cell survival by 70–90% were used (Fig. S7a, Table S9). To obtain the more comprehensive results, we compared cell viability data for all six cell lines combined (Fig. 4b). To discriminate growth factors’ ability to stimulate cell proliferation from their protective action against drugs, we compared growth factor-induced cell viability increase in drug-treated cells with mock-treated (DMSO) cells. EPO and NGF showed the most pronounced protective effects, while IGF-1 and HGF could not protect cells from any drug. We analyzed gene expression in NB cell lines from GSE78061 to determine which NB cluster each of these cell lines better represented based on the weighted mean squared expression of clusters DEGs. SH-SY5Y, Kelly, and SK-N-BE closer represented cluster 1, LAN-1 and SK-N-AS cluster 2, and SK-N-SH cluster 3 (Fig. S6b). Growth factors affected cell lines representing cluster 1 (SH-SY5Y, SK-N-BE, and Kelly) significantly stronger than other cell lines (Fig S6c). This is consistent with our results that NB tumors from cluster 1 overall have higher expression of receptors, including KIT, MET, NTRK2, and EPOR (Fig. 1b, Table S2). Overall we observed significant protective effects against all drugs except vincristine. However, EPO, NGF, and IGF-1 could protect individual cell lines from vincristine (Fig. S7b). These results are consistent with previous reports that EPO [36] and NGF [35] can protect NB cells from chemotherapy drugs, such as doxorubicin, etoposide, and vincristine.

To address whether such protective effect of these drugs can be attributed to changes in the expression level of receptor genes, we measured imatinib, dasatinib, crizotinib, and vincristine effects on *EPOR*, *IGF1R*, *KIT*, *MET*, *NTRK1*, and *NTRK2* expression. All drugs, except crizotinib, upregulated receptor gene expression in several cell lines (Fig. 4c). Changes in protein levels for EPOR and NGF receptor TrkA were confirmed by antibody staining (Fig. 4d). EPOR expression upregulation significantly correlated with EPO-induced cell survival (Fig. 4e), suggesting that receptor upregulation may be one of the possible mechanisms for cell death escape. However, this mechanism may be limited to EPO/EPOR signaling. To verify whether EPOR upregulation may be connected to NB cell survival, we downregulated EPOR expression in SH-SY5Y and LAN-1 cells using anti-EPOR shRNA (Fig. S8a). EPOR knockdown did not affect cell proliferation but significantly reduced EPO effect on cell survival for imatinib or dasatinib-treated cells (Fig. S8b).

### Multikinase inhibitors potentiate NB cells to EPO and NGF-mediated ERK activation

**Fig. 5:**
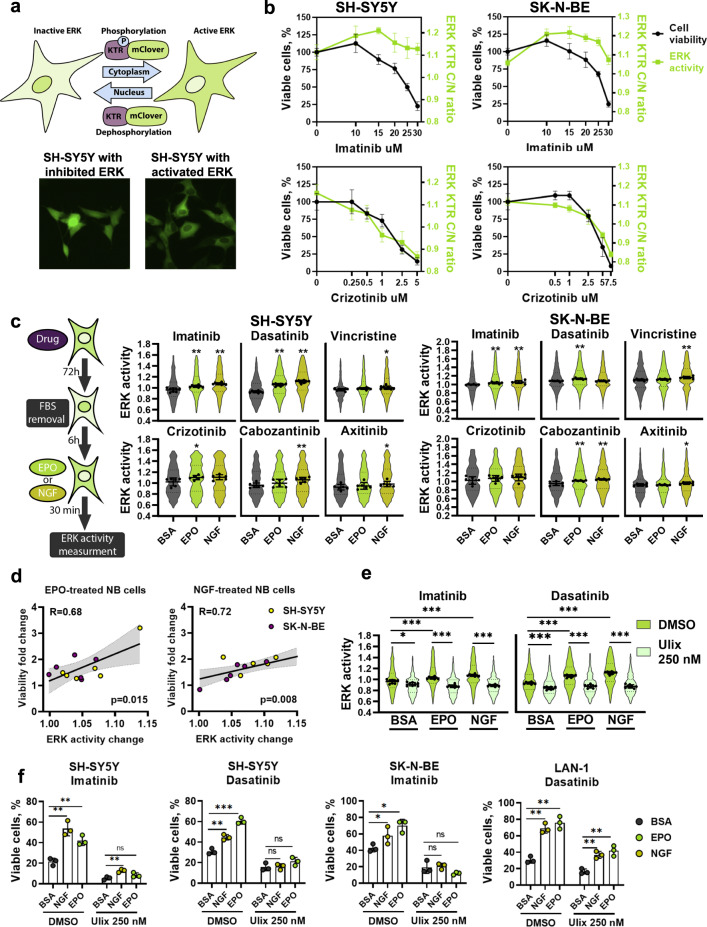
EPO and NGF protective action on NB cells are ERK-dependent. **a** Schematic presentation of ERK-KTR system. **b** ERK activity measured by ERK-KTR cytoplasm to nucleus ratio (C/N ratio) in SH-SY5Y and SK-N-BE 24 h after treatment and cell viability measured 72 h after treatment with imatinib and crizotinib. Data for other drugs are present in Fig. S10c. **c** SH-SY5Y, and SK-N-BE cells were treated with drugs (concentrations are listed in Table S9) for 72 h, and then the ability of EPO and NGF (100 ng/ml) to activate ERK was measured. Cells were serum-starved (by FBS removal) 6 h prior addition of recombinant proteins. BSA (0.1%), in which EPO and NGF were reconstituted, was used for a mock treatment. ERK activation by EPO and NGF in the absence of drug treatment is shown in Fig. S10a. Violin “superplots” [58, 59] show the average distribution of ERK activity (C/N ratio). Individual points on violin plots show median C/N ratios for each measurement (*n* = 6) of 150 cells on average. **d** Pearson correlation between EPO and NGF-induced changes in ERK activity and their effect on cell viability for SH-SY5Y and SK-N-BE cells treated with drugs. **e** SH-SY5Y cells were treated with imatinib (25 µM) or dasatinib (25 nM) for 72 h, and then the ability of EPO and NGF to activate ERK in the presence of ulixertinib (250 nM) was measured. Ulixertinib was added during serum starvation. Images were taken 30 min after addition of EPO or NGF, shown in Fig. S9. **f** Cell viability of SH-SY5Y, SK-N-BE, and LAN-1 cells after treatment with imatinib and dasatinib in combination with ulixertinib in the presence of EPO or NGF (100 ng/ml each). Cells were treated as described previously in Fig. 4. Additional controls are shown in Fig. S10b. Mean values, individual data points, and SD are shown on all graphs except violin plots. For violin plots, median values and 25 to 75th percentiles are shown. *p value < 0.05; **<0.01; ***<0.001 as calculated by Mann–Whitney *U* test.

Along with the growth factor signaling pathways, the MAPK cell survival pathway was strongly associated with poor therapy response (Fig. 2b). MAPK/ERK signaling is the central downstream signaling activated by growth factors and directly controls cell survival. We decided to investigate how two growth factors that showed the most prominent effects on cell survival (EPO and NGF) impact ERK activation during treatment with anticancer drugs. We employed the ERK-KTR (kinase translocation reporter) system that allows live-cell measurement of kinase activity in single cells and created SH-SY5Y and SK-N-BE cells with ERK-KTR [37, 38] (Figs. 5a, S9, S10a,b).

We observed different effects of drugs on ERK activity in NB cells. Treatment with imatinib, dasatinib, cabozantinib, and axitinib, but not crizotinib, maintained high ERK activity in NB cells, and ERK was not inhibited even at highly toxic concentrations (Figs. 5b, S10c). This is in line with our results that imatinib and dasatinib, but not crizotinib upregulated expression of growth factor receptors, which may contribute to maintaining ERK activity (Fig. 4c). When NB cells were treated with EPO or NGF after 72 h exposure to kinase inhibitors (Fig. 5c), these growth factors resulted in ERK activation (Figs. 5c, S9). Unlike NGF, EPO failed to activate ERK in non-treated cells (Fig. S10a). Notably, EPO and NGF-induced cell survival significantly correlated with ERK activation induced by them in drug-treated NB cell lines (Fig. 5d). To verify ERK involvement in NB cell survival, we tested ERK1/2 inhibitor FR180204 ability to affect ERK activation and cell survival induced by EPO and NGF. Since JAK/STAT is a canonical pathway activated by EPO, we also tested JAK2 inhibitor AG490. ERK inhibitor showed a much higher potential to inhibit EPO and NGF-induced ERK activation and survival in imatinib-treated cells (Figs. S10b, S11a). Then we validated these results using ERK1/2 inhibitor ulixertinib, which undergoes clinical studies for RAS mutated tumors, including NB (clinical trial NCT03698994) (Fig. 5e). To test if ERK inhibition would also block EPO and NGF’s protective action, we selected NB cells for which EPO and NGF protective effects were the highest and treated them with a combination of imatinib or dasatinib with ulixertinib (Figs. 5g, S11b). ERK inhibition completely blocked EPO and NGF in most cases for SH-SY5Y and SK-N-BE and sufficiently reduced EPO and NGF protective effect on LAN-1 cells.

### ERK1/2 inhibition enhances anticancer drugs action on NB cells

**Fig. 6:**
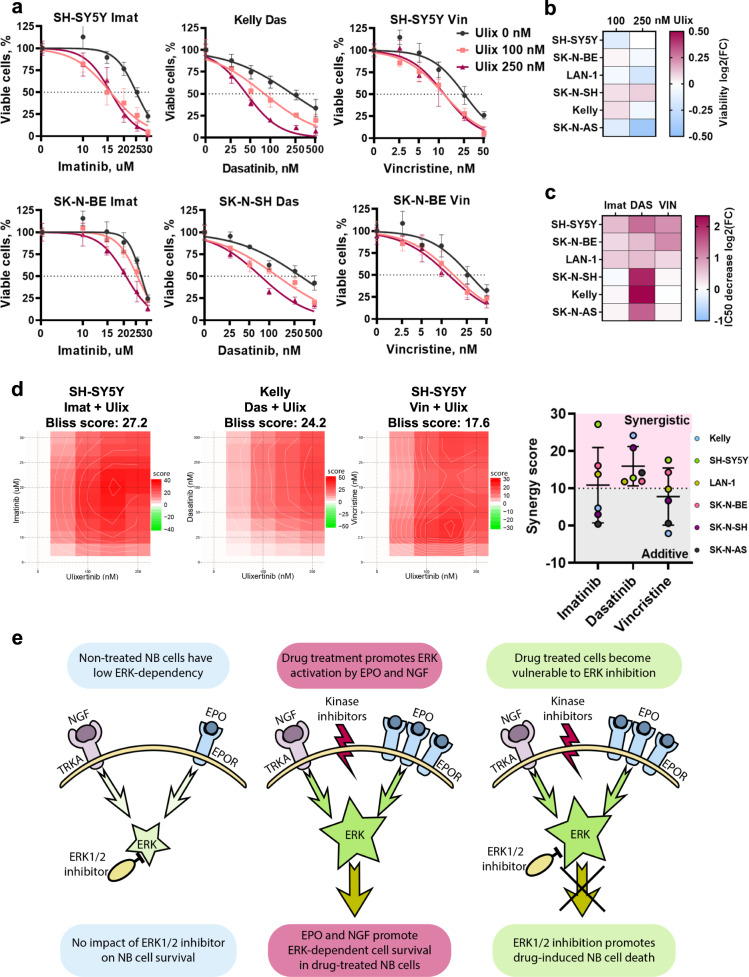
Analysis of synergistic action of anticancer drugs and ulixertinib on NB cell survival. **a** NB cell viability after treatment with imatinib (Imat), dasatinib (Das), or vincristine (Vin) in combination with ulixertinib (Ulix) for 72 h. For each ulixertinib concentration used in combination with other drugs, cell viability was normalized to viability of cells treated only with ulixertinib. Drug concentrations are shown on a logarithmic scale. DMSO was used for a control treatment. Two cell lines with the highest synergies are shown for each drug, additional graphs are provided in Fig. S13. **b** Heatmap showing ulixertinib induced toxicity when used in concentrations 100 and 250 nM without the addition of other drugs. **c** Heatmap for imatinib (IM), dasatinib (DAS), and vincristine (VIN) IC50 decrease when drugs were used in combination with 250 nM ulixertinib compared to control treatment with DMSO. Exact values are provided in Table S10. **d** Heatmaps show drugs synergy as calculated by the Bliss method using SynergyFinder2.0 for cell lines with the highest scores for each drug. Dot plot shows synergy scores for each drug combination with ulixertinib and each cell line. Mean synergy values for each drug and SD are shown. Synergy scores were calculated using SynergyFinder2.0 [47]. **e** Schematic representation of ERK-dependent NB cell adaptation to multikinase inhibitors.

We noticed that ERK1/2 inhibition promoted cell death induced by imatinib and dasatinib, and treatment with these drugs maintained high ERK activity level. To investigate this, we first compared the combined action of imatinib with either ERK1/2 or JAK2 inhibitor on a panel of six NB cell lines (Fig. S12a). ERK1/2 inhibitor FR180204 combined with imatinib showed more synergistic action on NB cell death than JAK2 inhibitor AG490 and significantly inhibited NB cells’ long-term proliferation in the presence of imatinib (Fig. S12b). We expanded these experiments and tested the combined action of ERK inhibitor ulixertinib with imatinib, dasatinib, or vincristine on a panel of six NB cell lines (Figs. 6a, S13). Ulixertinib in the concentration of 250 nM showed significant toxicity only for SH-SY5Y (35% decrease in viability) and SK-N-AS cells (25% decrease in viability), while 100 nM concentration was not toxic for all tested cells (Fig. 6b). The addition of ulixertinib lowered IC50 values for tested drugs, and the most pronounced effects were observed for dasatinib (on average 2.9-fold decrease in IC50) (Fig. 6c, Table S10). Ulixertinib displayed high synergy with dasatinib (6/6 cell lines with synergy score >10) and moderate synergy with imatinib (3/6) and vincristine (2/6) (Fig. 6d). Based on our data, we speculate that activation of EPO and NGF signaling is a compensatory response of NB cells to multikinase inhibitors that promote NB cell survival in an ERK-dependent manner (Fig. 6e).

## Discussion

Our study shows that the expression of cellular receptors and their downstream kinases can define NB subtypes which have a distinct association with patient survival. We constructed a model for predicting patient survival based on 48 genes expression, which yields consistent results for different datasets. We show that this model can be used to discriminate patients with stage 4 tumors without MYCN-amplification into patients, for whom current treatment will work well with survival >90% and for whom standard therapy will probably fail with survival probability <10%. This indicates that measuring the expression of these 48 genes in addition to standard NB diagnosis and risk assessment could be beneficial for predicting therapy efficacy, especially for stage 4 tumors without MYCN amplification. Also, our gene panel includes many drug targets, such as ALK, KIT, MET, RET, FGFR2, and FLT1, which can be potentially used to select alternative treatment options.

We identified EPOR signaling and ERK activation as possible critical modulators of NB progression and therapeutic resistance. Although EPO is considered a hematopoietic growth factor, its role in the neuronal cells maintenance has been previously documented [39]. One histological study showed that EPOR is expressed in more than 70% of NB tumors and has higher expression in lymph node metastases than the primary tumor, but high EPOR protein expression was associated with favorable outcomes for NB patients [40]. Our data and meta-analysis of published datasets strongly argue with the latter report [40] and are in line with most of the other studies on this topic, including involvement of EPO in NB [36] and breast cancer cell survival [41]. Taking together our results that EPO-mediated survival correlates with EPOR expression upregulation by kinase inhibitors, an increase of EPOR expression in relapsed tumors, and that EPO is produced by NB [42] and neural crest cells [43] in more than 30% NB tumors [40], we propose that *EPOR* upregulation is a potential marker for NB tumors, which might escape anticancer therapy and result in a relapse. Interestingly, we found significant correlation between *EPOR* expression and patient survival only for MYCN non-amplified tumors. The possible reason for this is lower *EPOR* expression in MYCN amplified than in non-amplified tumors, and MYCN amplification been stronger prognostic factor than *EPOR* expression. We did not find any difference in EPO action on survival of MYCN amplified and non-amplified NB cells. However, the exact prognostic significance of EPOR as such marker should be further investigated in vivo experiments and clinical trials.

Notably, we discovered a correlation between ERK activation and EPO or NGF protective action and the direct effect of ERK inhibition on NB cell survival. A likely mechanism for EPO protective action on NB cells is a combination of a dynamic upregulation of EPOR expression and the selective pressure for NB cell subsets expressing EPOR. Another potential mechanism for NB drug resistance is that many kinase inhibitors can be targets for MDR1 protein, but we did not observe any significant changes in expression of its gene *ABCB1* after EPO or NGF treatment or ERK inhibition (Fig. S8c). Currently, ERK or MEK inhibitors are considered mainly for the treatment of NB tumors with activating RAS mutations, which constitute a very aggressive NB subtype, but are relatively rare (<1% of primary tumors) [5, 44]. Our data indicate that ERK inhibitors might improve the outcome for many NB tumors, primarily in which survival depends on growth factor signaling rather than mutations in the RAS/MAPK pathway.

Our study has several limitations: most NB tumors used for transcriptomic analysis were treated with the standard treatment protocols (such as NB2004), which do not include kinase inhibitors. However, we and other groups report that EPO and NGF can protect NB cells from vincristine, etoposide, and doxorubicin EPO [35, 36], which are used for NB treatment. Here we show that growth factors, such as EPO, NGF, and BDNF, exert a significantly stronger protective effect against kinase inhibitors than against vincristine, and these growth factors may potentially hinder therapy efficacy of kinase inhibitors. Another limitation is that our analysis of growth factor signaling in NB tumors is based on gene expression rather than receptor and kinase phosphorylation. However, main goal of our study was to test whether gene expression signatures could be used to determine NB outcome prognosis and growth factors that may functionally affect NB cells. Results obtained from transcriptome analyses were confirmed on a panel of NB cell lines. Evaluating signaling in patient or xenograft tumors on protein level could further improve our mechanistic understanding of how exactly cells adapt to the therapy. Although combinations of ERK inhibitor ulixertinib with imatinib and dasatinib were tested only on cell lines, all these drugs were previously tested in NB xenograft models, and clinically relevant concentrations can be achieved. The drug concentrations, except for axitinib, used in our study are similar or only slightly higher than the observed serum concentrations in treated humans (Table S1). Importantly, dasatinib, imatinib, crizotinib, and ulixertinib have been tested in NB clinical trials, and their safety and clinical potential have been verified.

In conclusion, our results indicate that assessing growth factor signaling in NB tumors can lead to the development of novel risk assessment strategies and treatment options, and a better understanding of therapy resistance mechanisms. We show that ERK inhibition is a potential therapeutic approach to enhance the efficacy of anticancer drugs used to treat NB tumors and not limited to NB tumors with RAS mutations.

## Materials/subjects and methods

### Cell cultures, inhibitors, and growth factors

Human NB cell line: SH-SY5Y, SN-K-AS, SK-N-SH, LAN-1, SK-N-BE, and Kelly were cultured in RPMI 1640 medium (Gibco) supplemented with 10% fetal calf serum (FCS) 100 U/ml penicillin, 100 μg/ml streptomycin, and 1 mM sodium pyruvate at 37 °C and 5% CO_2_. Cell densities used for experiments and mutation status for each cell line is provided in Table S1. HEK293T cells were used for generation of lentiviral particles stocks and were cultured in DMEM medium (Gibco). All NB cell lines were gifted by the Heinrich-Pette Institute—Leibniz Institute for Experimental Virology. HEK293T were gifted by Boris Fehse and Kristoffer Riecken from University Medical Center Hamburg-Eppendorf, Hamburg, Germany. None of the used cell lines is listed in the list of commonly misidentified cell lines maintained by the International Cell Line Authentication Committee. List of drugs and growth factors used in this study is provided in Table S1.

### Lentiviral pseudotyped particles production and titration

pLentiCMV Puro DEST ERKKTRClover was a gift from Markus Covert (Addgene plasmid # 59150) [37]. The stocks containing VSV-G pseudotyped lentiviral particles were generated by co-transfection of HEK293T with LeGO-C shRNA or pLentiCMV Puro DEST ERKKTRClover, and packaging plasmids. For the creation of SH-SY5Y and SK-N-BE cells expressing ERK-KTR, cells were transduced with ERKKTRClover lentiviral particles to achieve ~30–50% transduction rate and then transduced cells were selected with medium supplemented with 1 μg/ml puromycin (Sigma). The DNA sequences encoding anti-EPOR (shEPOR) or non-specific control (shSCR) shRNAs were subcloned into the HpaI/XhoI sites of LeGO-C plasmid containing the mCherry protein. LeGO-C plasmid for shRNA expression was a gift from Boris Fehse (http://www.lentigo-vectors.de/) [45]. shEPOR sequences are provided in Table S1, shSRC is described in [46]. The amount of shRNA lentiviral particles per cell that resulted in at least 90% SH-SY5Y and LAN-1 cells transduction was used for further experiments. Transduction rates were verified by flow cytometry.

### Analysis of cell survival, drugs IC50, and synergy

The number of viable cells was counted in Neubauer chamber by trypan blue exclusion method. For growth factor-mediated cell survival (Fig. 4b) cell viability was measured by XTT (Sigma, USA). For cell viability calculation measured by XTT absorption signals for growth media without cells were subtracted from signal for wells containing cells. Signal for mock-treated cells (treated with DMSO) was considered as 100%. For calculating drugs synergies or IC50 values cells were treated with drug combinations for 72 h. For each ERK or JAK2 inhibitor concentration used in combination with other drugs cell viability was normalized to the number of cells treated without additional drug. Approximations for IC50 calculations were performed by nonlinear regression with variable slope (four parameters) and robust fitting. Synergy scores were calculated using Bliss model in SynergyFinder2.0 [47].

### Direct flow cytometry

For TrkA detection we used FITC-conjugated anti-TrkA antibodies (ab194321, Abcam, USA) and for EPOR detection we use primary anti-EPOR antibodies (PA5-8484, ThermoFisher Scientific, USA) and secondary anti-rabbit Ig antibodies conjugated with FITC (F4890, Sigma, USA). Secondary antibodies were used as a control for non-specific staining. Measurements were performed on LSRFortessa flow cytometer (BD Biosciences) and analyzed with FlowJo software.

### Quantitative real-time RT-PCR

Real-time RT-PCR was performed using the Maxima SYBR Green Supermix (Thermo Scientific, USA) and CFX96 Real-Time System (Bio-Rad, USA). The expression levels of studied genes were normalized to that of the human *GAPDH*, Ct values, and relative expression was determined by CFX Manager 3.1 software (Bio-Rad, USA). Primer sequences are presented in Table S1. All PCR measurements were performed two independent times each in triplicate.

### Neuroblastoma tumor biosamples

For this study, we used 60 experimental formalin-fixed, paraffin-embedded NB tissue samples with tumor cell content exceeding 70%, obtained from 60 patients treated at the D. Rogachev Center of Pediatric Hematology, Oncology and Immunology (CPHOI), Moscow. For all the biosamples, informed written consents to participate in this study were collected from the patient’s representatives. The consent procedure and the design of the study were approved by the ethical committees of the CPHOI and of the Engelhardt Institute of Molecular Biology. Tumors were evaluated by a pathologist to confirm the diagnosis and estimate the content of tumor cells. All patients were treated according to NB2004 protocol. “No response”, relapses and partial response to the therapy outcomes were considered as poor response, and others were considered as good response outcomes. The response was determined based on tumor progression after each round of chemotherapy. All patients were monitored for at least 1 year; the median observation time was 817 days. Transcriptomic data for 41 patients were previously published and deposited to GEO with accession number GSE96629 [28], we updated clinical information for these data and provide additional 19 tumor samples using the same accession number GSE96629.

### Identification of differentially expressed genes and calculation of molecular pathway activation level

Synthesis of microarrays, library preparation, and hybridization was performed as described previously [28]. Gene expression data was obtained by using customized microarray platform, covering 3706 human gene transcripts involved in carcinogenesis. Gene expression data were normalized using quantile normalization [48]. Differential gene expression analysis was performed in two ways: (1) responding patients (43 samples) vs. non-responding patients (17 samples) and (2) metastatic patients (30 samples) vs. patients without metastasis (30 samples). Significance of gene expression difference was assessed using two-sided *t* test with a *p* value threshold of 0.05. Based on normalized gene expression values of these DEGs we calculated pathway activation levels for 380 molecular pathways extracted from Pathway Central database (https://geneglobe.qiagen.com/ru/explore/). For each molecular pathway we calculated pathway activation level using previously described formula [29]. For each molecular pathway we calculated pathway activation level using the following formula [29]:

$$PALp \,=\, {\sum} {NIInp \,\times\, ARRnp \,\times\, \log CNRn} {\sum} {NIInp \,\times\, |ARRn|n/n}$$

where PALp—molecular pathway p activation level; CNRn (case-to-normal ratio)—ratio of the protein-encoding gene n product concentrations in the test sample and in the norms (average value in the control group); NIInp—index of gene product n assignment to the pathway p, assuming the values equal to 1 for gene products included in the pathway and equal to 0 for gene products not included in the pathway; discrete value ARRnp (activator/repressor role) is deposited into the molecular pathway base and determined for a gene n in the pathway p as follows: $ARRnp$ = {−1;protein $n$ is a signal repressor in a pathway $p$ − 0.5; protein $n$ is more likely a signal repressor in a pathway $p$0; the role of a protein $n$ in a pathway $p$ is either ambivalent or neutral 0.5; protein $n$ is a signal activator in a pathway $p$1; protein $n$ is a signal activator in a pathway $p$.

For each pathway we then performed non-paired *t* test in order to estimate differential activation of this pathway between: (1) responding vs. non-responding patients and (2) metastatic vs. non-metastatic patients. We adjusted the calculated *p* values for multiple hypotheses testing using FDR method and set the threshold 0.05. Heatmap for PAL visualization was created using ClustVis web tool (https://biit.cs.ut.ee/clustvis/) [49].

### UMAP and cluster DEGs

Initially we selected nine datasets from R2: Genomics analysis and visualization platform with available overall patient survival data (Table S1). For each dataset each gene expression data was normalized as fold change from the mean expression of this gene in a dataset. Four datasets were removed: TARGET due strong clustering based on dataset origin and Oberthuer [50] having significantly lower gene representation than other datasets, SEQC [51] and Cangelosi [27] due to having same patient samples as other datasets (Kocak for SEQC, Kocak, TARGET, SEQC, and Versteeg for Cangelosi). We used gene expression data for 1226 NB tumors from five datasets: Kocak, NRC, Versteeg, Maris, and Westermann [18, 20, 22, 23, 52] (Table S1) and a set of 168 growth factor-related genes present in all datasets (Table S2). Tumor clustering was performed by UMAP dimension reduction [53] and HDBSCAN density-based hierarchical clustering [54] algorithms using following Python libraries: https://github.com/lmcinnes/umap and https://github.com/scikit-learn-contrib/hdbscan. We used the following parameters for UMAP: n_neighbors = 50, min_dist = 0, and correlation metric; and for HDBSCAN: min_samples = 50, min_cluster_size = 100. To identify tumor clusters we employed density-based hierarchical clustering using HDBSCAN. 37 outliner samples were removed from the analysis. Differentially expressed genes for tumors in clusters against all over tumors were identified by multiple Mann–Whitney tests with FDR correction using SciPy (https://github.com/scipy/) and statsmodels (https://www.statsmodels.org/) Python libraries.

To identify which cluster a particular cell line represent we calculated scores for each cluster based on expression of differential genes in a particular cell line. We normalized gene expression data for 26 NB cell lines from Russel dataset (Table S1) in the same way as for NB tumors. Then we used gene expression data for SH-SY5Y, SK-N-BE, LAN-1, SK-N-AS, SK-N-SH, and Kelly cells to calculate scores based on the following formula:

$$S \,=\, \mathop {\sum}\limits_i {{\it{log}}_2\left( {exp_i} \right) \,\ast\, c_i}$$

*S*—cluster score for a cell line, exp_i_—expression of i differential gene for a cell line, *c*_i_—coefficient, which equals 1, if this gene is upregulated in a cluster, −1 if is downregulated.

Cell lines were assigned to a cluster with the highest score.

### Creation of survival prediction model

For training and testing our prediction model we selected the integrated and batch-controlled dataset, which contains survival and gene expression data for 786 NB tumors [27]. Dataset was normalized in the same way as described for UMAP. This dataset was divided into 153 MYCN amplified and 629 MYCN non-amplified tumors, four samples were excluded due to unknown MYCN status. Each group was randomly divided into train (70%) and test (30%) datasets. For the first training round we selected 147 genes which are differentially expressed in clusters 1–3 for MYCN amplified or MYCN non-amplified tumors. To create quantitative model for survival prediction based on gene expression we used logistic regression with elastic net regularization:

$$p\left( z \right) \,=\, \frac{1}{{1 \,+\, e^{ - z}}}$$

$$z \,=\, y_o \,+\, \mathop {\sum}\limits_i {\omega _i \,\ast\, \exp _i}$$

$$\hat \omega \,=\, \mathop {{\min }}\limits_\omega \left( {\left\| {z \,-\, X\omega } \right\|_2 \,+\, \alpha \,\ast\, l1 \,\ast\, \left\| \omega \right\|_1 \,+\, \alpha \,\ast\, \frac{{\left( {1 \,-\, l1} \right)}}{2} \,\ast\, \left\| \omega \right\|_2} \right)$$

Where p(z)—survival probability function, exp_i_—expression of gene i, *ω*_i_—weight of gene i, *y*_0_—intercept, $\hat \omega$—weights estimator, *X*—expression matrix, *ω*—weights matrix, *α*, and l1—elastic net penalty parameters.

Optimal logistic regression and elastic net regularization parameters were selected by fivefold cross validation, and then gene weights in the model were calculated. Parameter optimization and model calculation were performed separately for MYCN amplified and non-amplified tumors. For the second training round for each model we selected from 10 to 50% genes based on highest absolute weight values. Second round training was performed in the same manner as the first round. Optimal amount of genes for the second model was determined by F1 model parameter for test datasets. Final model computes survival probability using parameters for MYCN amplified or non-amplified tumors depending on tumor MYCN status. If status is unknown than MYCN non-amplified parameters are used. Before application of prediction model to other datasets they were initially normalized in the same way. Logistic regression was performed in Python 3.8 using scikit-learn library.

### Gene set prognostic scoring (GPScore) and multidimensional scaling

We used GSEA v.4.03 (http://software.broadinstitute.org/gsea/downloads.jsp) to identify enriched gene sets associated with gene expression in NB patients. Enrichment results satisfying a nominal *p* value <0.05 with a false discovery rate (FDR) < 0.25 were considered statistically significant. Top 100 enriched GO gene sets (based on higher normalized enrichment score) were selected from GSEA for EPOR and KIT. For multidimensional scaling of enriched GO gene sets, we used a web interface REVIGO [55]. SimRel score for depth of GO gene annotation was used for multidimensional scaling and the obtained results were then plotted in Cytoscape software [56]. Gene set prognostic score was calculated according to the following formula:

$$GPScore \,=\, \frac{{N_u}}{{N_u \,+\, N_f}}$$

where *N*_*u*_—a number of genes in a set those high expression is associated with unfavorable prognosis for NB patients; *N*_*f*_—a number of genes in a set that high expression is associated with favorable outcome. Versteeg (*n* = 88) (GSE16476), NRC (*n* = 283) (GSE85047), and Kocak (*n* = 476) (GSE45547) datasets were used for estimation of gene expression correlation with prognosis, since these datasets have available survival data, similar tumor stage and types distribution, and gene presentation. Kaplan–Meier nonparametric estimator from R2: Genomics analysis and visualization platform (http://r2.amc.nl) was used to determine association of high gene expression with overall survival. Only genes that have statistically significant correlation (*p* < 0.05 after Bonferroni correction) were considered as associated with prognosis. 20 random gene sets (from 50 to 1000 genes) were analyzed to determine baseline prognostic score values for each dataset. These values were used to identify gene sets with prognostic scores that differ from baseline scores in all three datasets. For each gene set prognostic scores for three dataset were compared with mean prognostic scores for 20 random gene sets using two-sided *t* test. Corrections for multiple comparisons were performed using false discovery rate (FDR), with *q* values < 0.01 considered as statistically significant. Circos plots were created using Circos software (http://circos.ca).

### Mutation analysis and drug selection

To analyze recurrent mutation in NB patients and cell lines frequencies we used TARGET dataset [5] and CCLE database [32] (Table S1). Silent mutations were excluded from the analysis. Mutations frequency (rate) was calculated as a number of patients or cell line with mutated gene divided by a total number of patients or cell lines present in a dataset. To identify drugs which may directly target one or more selected proteins we used DSigDB database and an algorithm which allows finding drugs by their potential targets [33].

### ERK-KTR quantification

For nuclear segmentation cells were incubated with 500 ng/ml Hoechst-33342 for 30 min before imaging. In experiments with growth factors cells were starved in medium without serum for at least 6 h before addition of growth factors. Each experiment was repeated at least two times, two microscopic fields with appropriate densities were chosen for imaging for each well. Cytoplasm to nucleus ratios (C/N ratio) of mClover intensity were calculated for each cell. Illumination correction, segmentation, and object intensity calculations were performed with CellProfiler [57]. Median intensities of mClover fluorescence in cytoplasm and nucleus were quantified and used to calculate cytoplasm to nucleus (C/N) ratios for each cell. C/N ratios were normalized: 1 represents maximum observed ERK activity in individual cell and 0—minimal ERK activity. All images were obtained by Leica DMI8 automated microscope and EVOS FL using ×10 magnification lenses. Data processing was performed in Python and GraphPad Prism 9. Violin plot for C/N ratios were made using the “superplots” concept for visualization of cell-to-cell and sample-to-sample variance [58, 59].

### Statistical analysis

All the data are expressed as mean ± SD from at least three individual experiments, unless stated otherwise in the text. Statistical significances of differences observed in cell viability experiments were determined by Mann–Whitney nonparametric test. Statistical significances for real-time PCR experiments were determined by unpaired two-sided Student *t* test. Kaplan–Meier estimation was performed using R2: Genomics analysis and visualization platform. Statistical calculations were performed in Python 3.7 and GraphPad Prism 9 software.

## Supplementary information

Supplemental material

Table S1

Table S2

Table S3

Table S4

Table S5

Table S6

Table S7

Table S8

Table S9

## Data Availability

The source codes used in this study are available at GitHub https://github.com/CancerCellBiology/Lebedev-et-al-NB-EPO-.
