# Supplementary material for: Growth factor signaling predicts therapy resistance mechanisms and defines neuroblastoma subtypes
Source: Oncogene. 2021 Sep 23;40(44):6258–72. doi: 10.1038/s41388-021-02018-7 (PMC8566230; doi:10.1038/s41388-021-02018-7)
Supplement: Supplementary file 1 — Supplemental material [file 41388_2021_2018_MOESM1_ESM.docx]

**
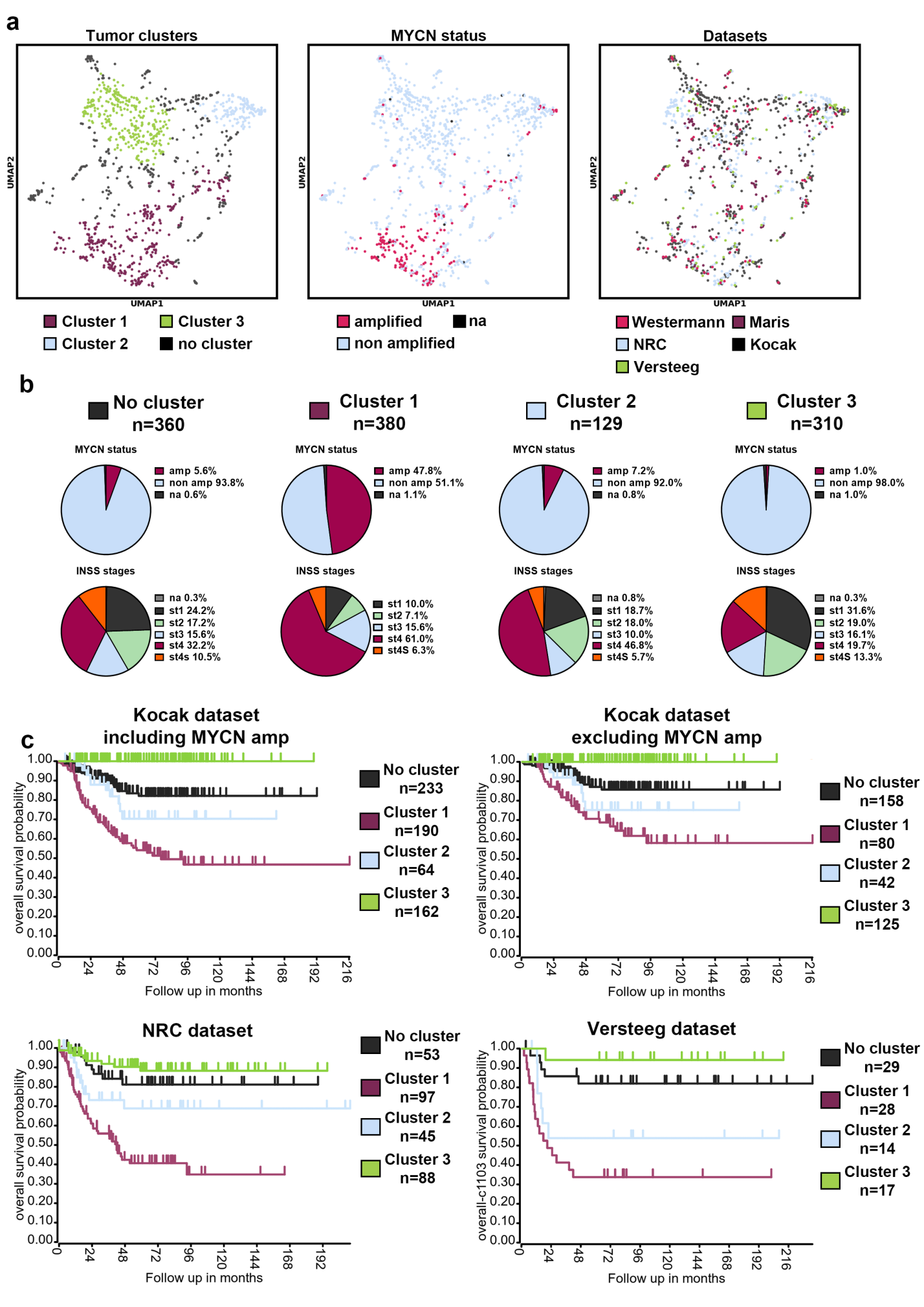
**

**Figure S1. UMAP results for NB tumors based on growth factor-related gene expression. (a)** Clusters identified with HDBSCAN, MYCN status and datasets used are marked for each tumor. **(b)** Distribution of MYCN amplified tumors and INSS stages in each cluster. **(c)** Kaplan Meier overall survival analysis for patients from identified clusters in the Kocak dataset (including and excluding MYCN amplified tumors), and in NRC and Versteeg datasets (all tumors included).

**
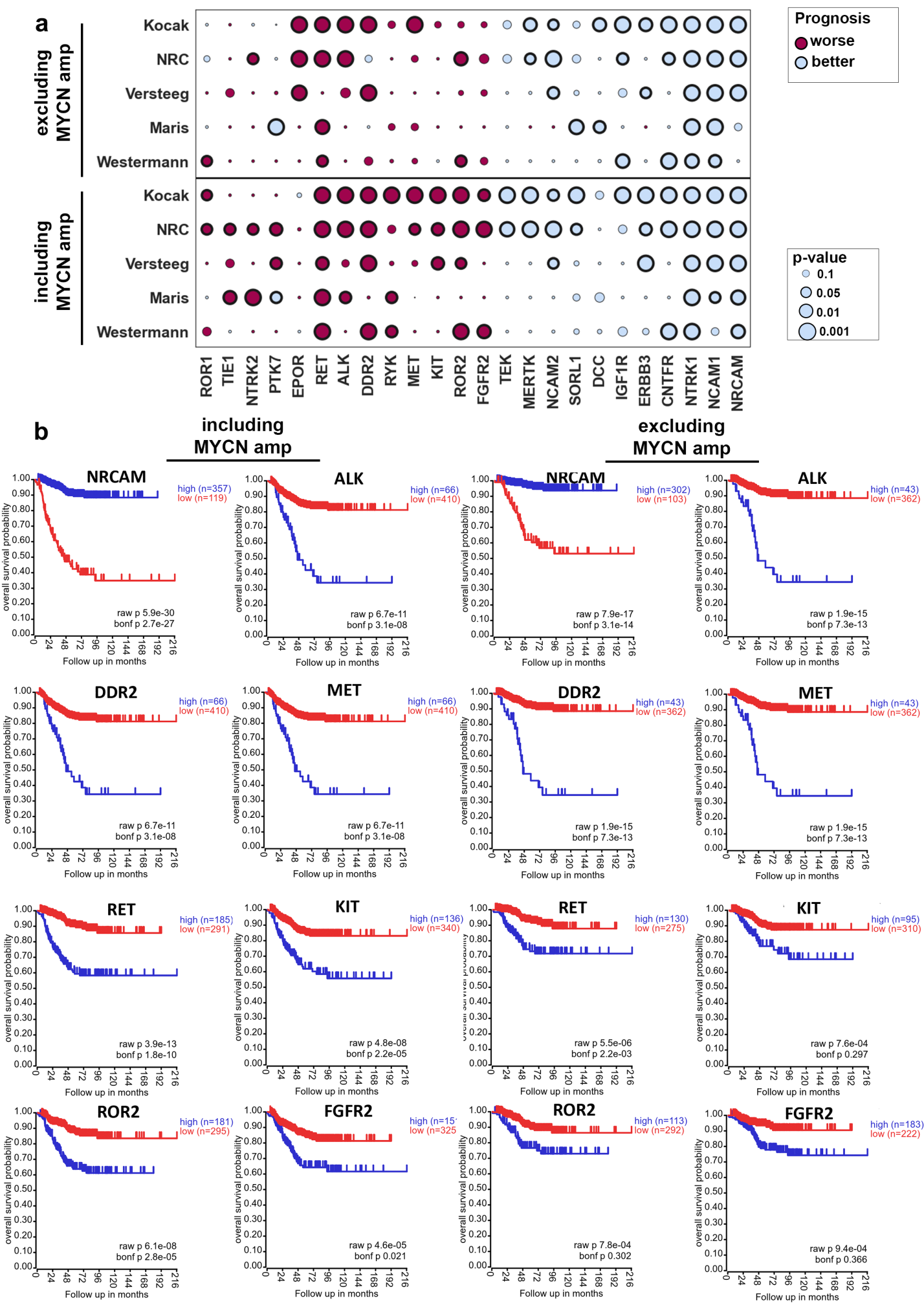
**

**Figure S2. (a)** Association of receptor gene expression with patient overall survival prognosis in NB datasets with included or excluded MYCN amplified tumors. The color indicates whether high gene expression is associated with worse or better prognosis. Genes are ordered based on Ward hierarchical clustering. Kaplan Meier scanner from the R2 platform was used for survival analysis, p-values are provided after Bonferroni correction. Significant associations (p<0.05) are highlighted by bold borders. Only genes with p<0.05 in at least two datasets when MYCN amplification excluded or included are shown. Exact p-values are provided in Table S3. **(b)** Kaplan Meier overall survival analysis based on gene expression for patients in the Kocak dataset (including and excluding MYCN amplified tumors).

**
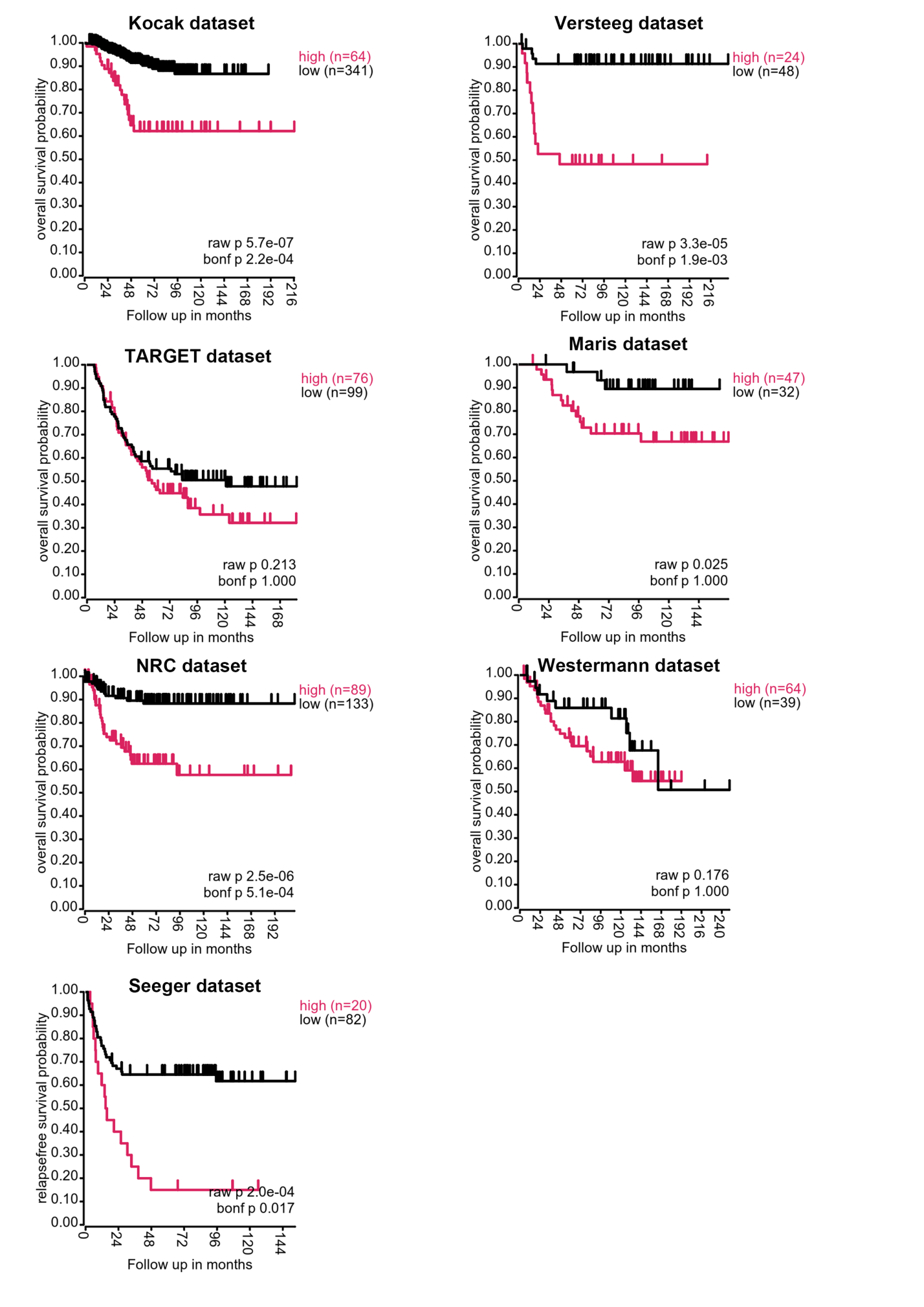
**

**Figure S3. Association of EPOR with NB outcome.** NB patient overall and relapse-free survival with high or low *EPOR* expression provided for different NB datasets from R2: Genomics analysis and visualization platform ([http://r2.amc.nl](http://r2.amc.nl/)) (Table S1)**.**

**
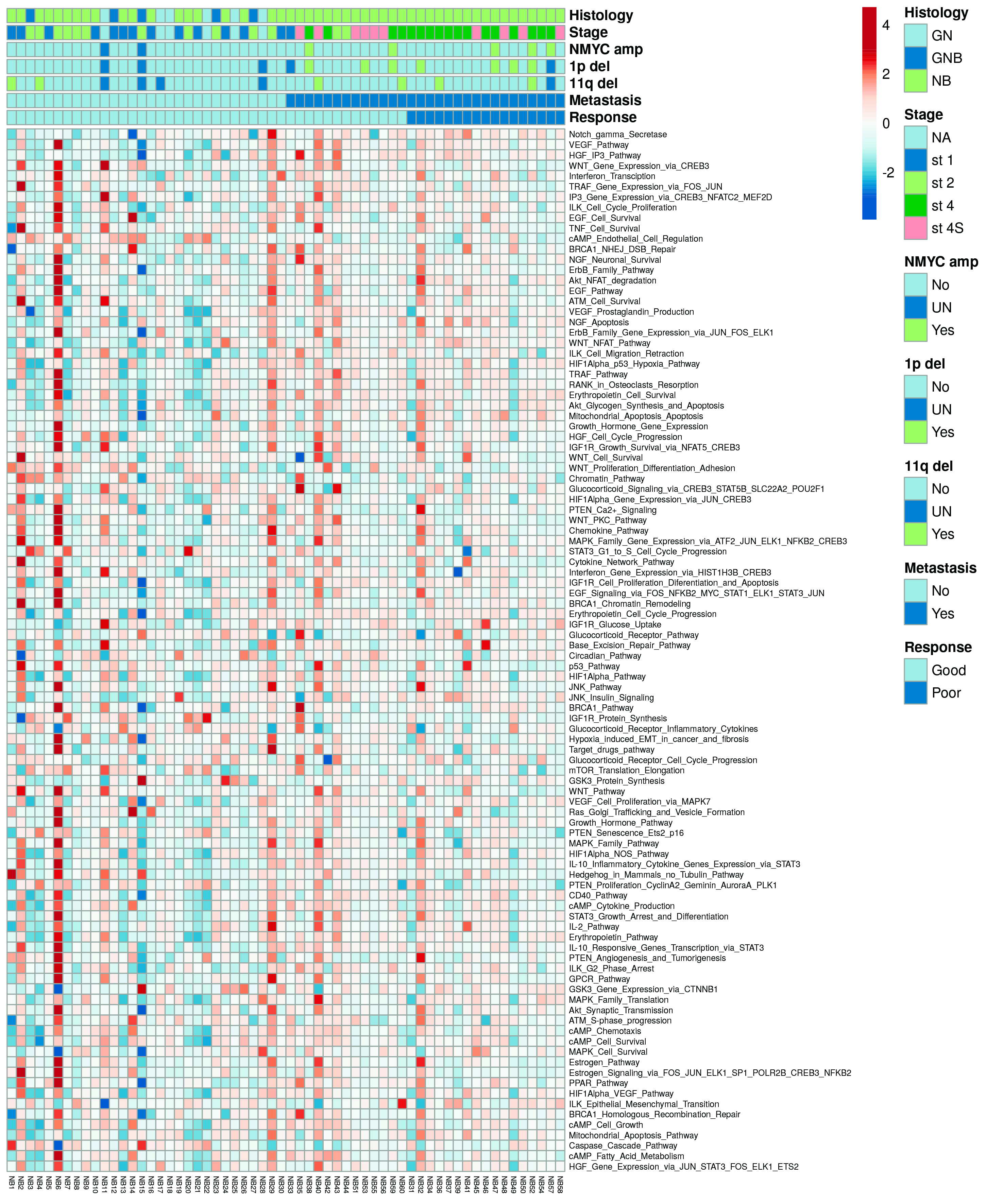
**

**Figure S4. Heatmap of signaling pathways activation in NB tumors.** Heatmap of signaling pathways activation in 60 neuroblastoma (NB), ganglioneuroblastoma (GNB), and ganglioneuroma (GN) tumors. Top 100 pathways that differentially activated/downregulated in metastatic vs. localized NB tumors that were selected based on FDR p-value. Heatmap was created using ClustVis web tool (https://biit.cs.ut.ee/clustvis/).

**
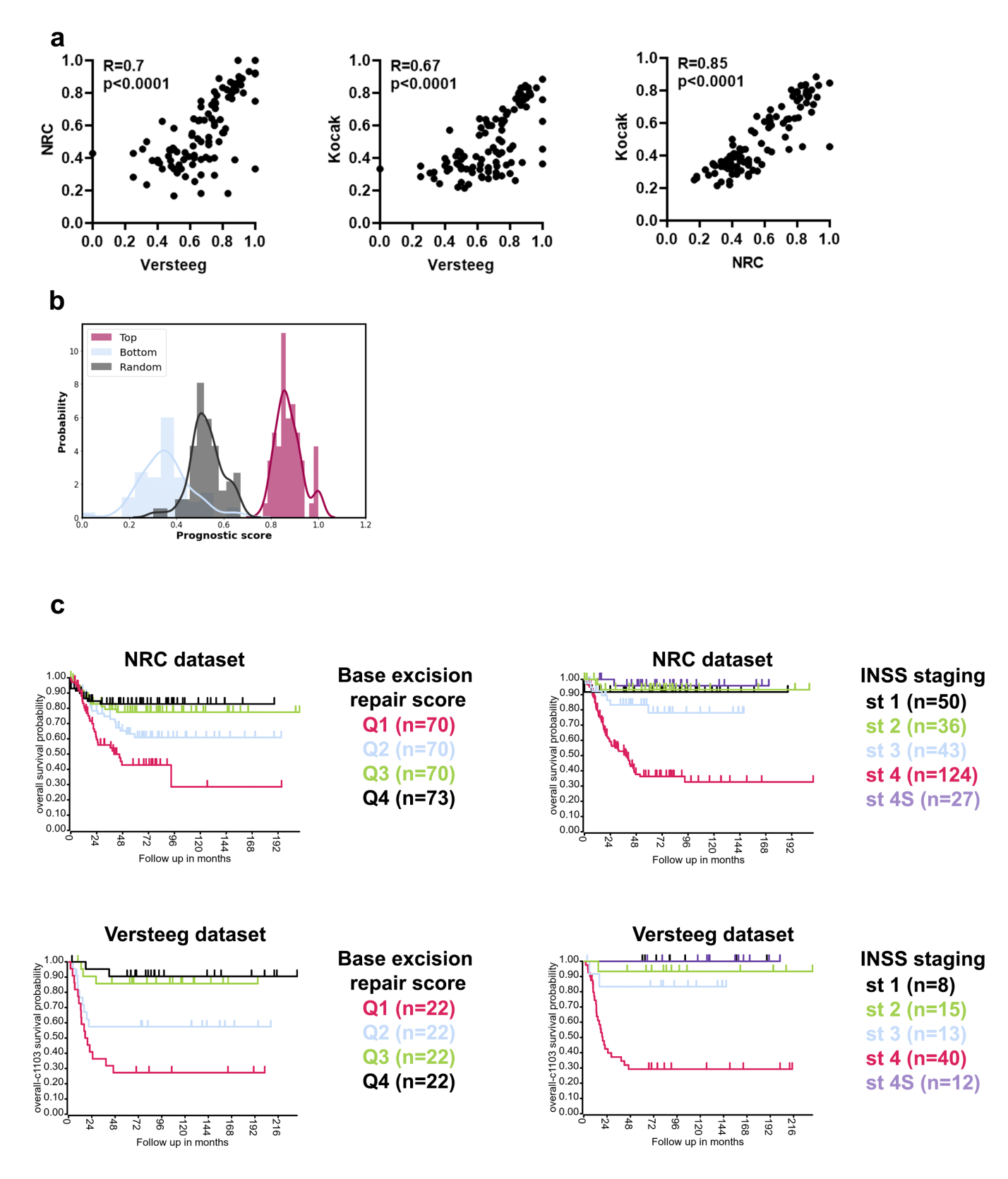
**

**Figure S5. Verification of Gene set Prognostic Scoring (GPScore) method. (a)** Prognostic scores Spearman correlations calculated for top 100 enriched gene sets for EPOR in three different NB datasets. Each point represents individual gene set. **(b)** Distribution of prognostic scores for 20 gene sets with highest prognostic scores for EPOR (Top), 20 gene sets with lowest prognostic scores for EPOR (Bottom), and 20 randomly generated gene sets (Random). **(c)** Kaplan-Meier survival analysis for NRC and Versteeg datasets based on base-excision repair score (divided in quartiles from highest Q1 to lowest Q4 scores) and INSS stages.

**
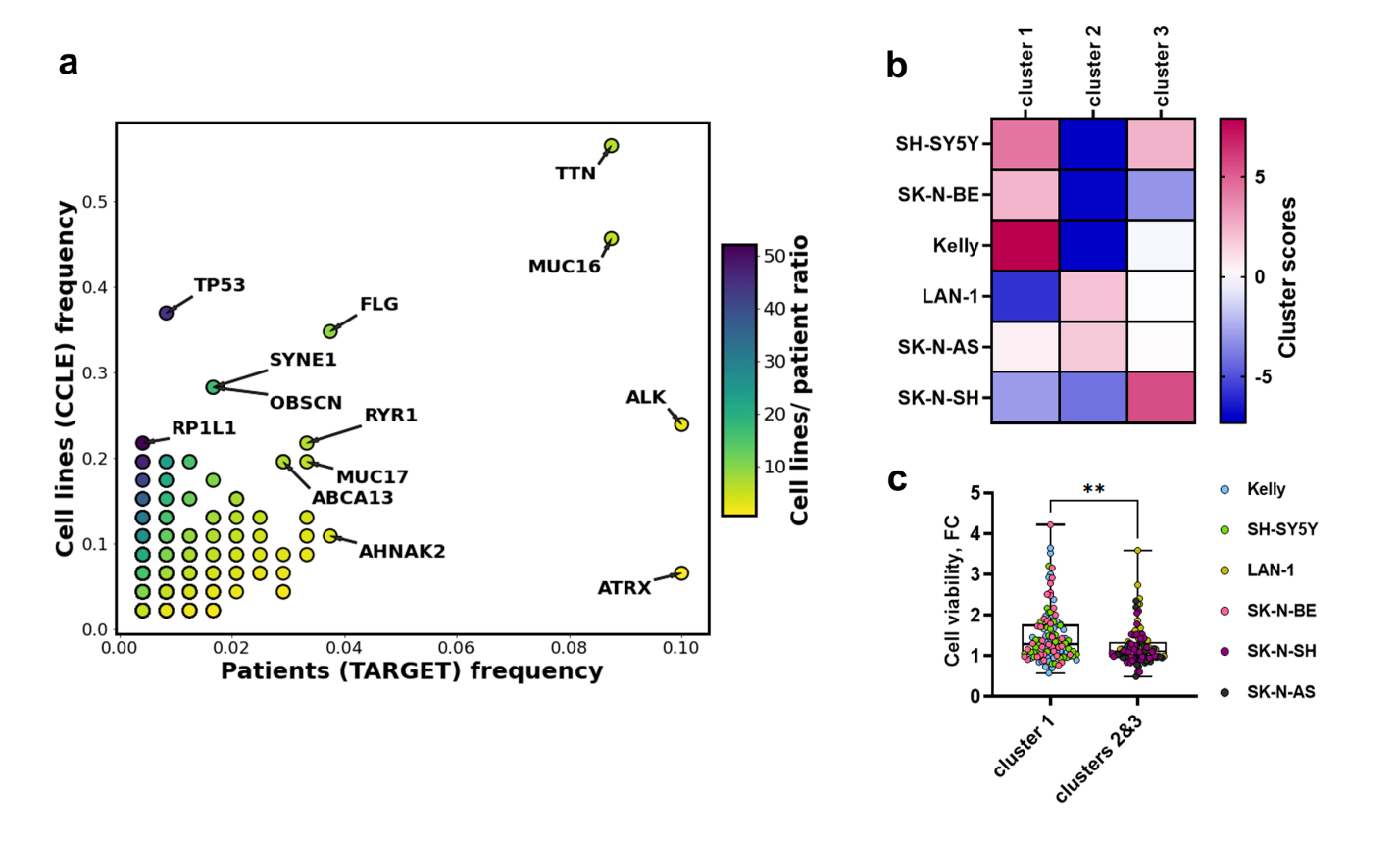
**

**Figure S6. Common mutations and expression features for NB cell lines. (a)** Mutations frequencies in cell lines (CCLE dataset) and in NB tumors from TARGET dataset. Color indicates the ratio between mutation frequencies observed for cell lines and for NB patients. **(b)** Heatmap showing calculated “cluster scores” for each cell line using gene expression data from Russel dataset. **(c)** Comparison of growth factor effects on cell survival during drug treatment for cells belonging to cluster 1 vs. clusters 2 and 3. Dots represent results for each cell line treated with one of the six anti-NB drugs. **- p-value< 0.01 as calculated by Mann-Whitney non-parametric test.


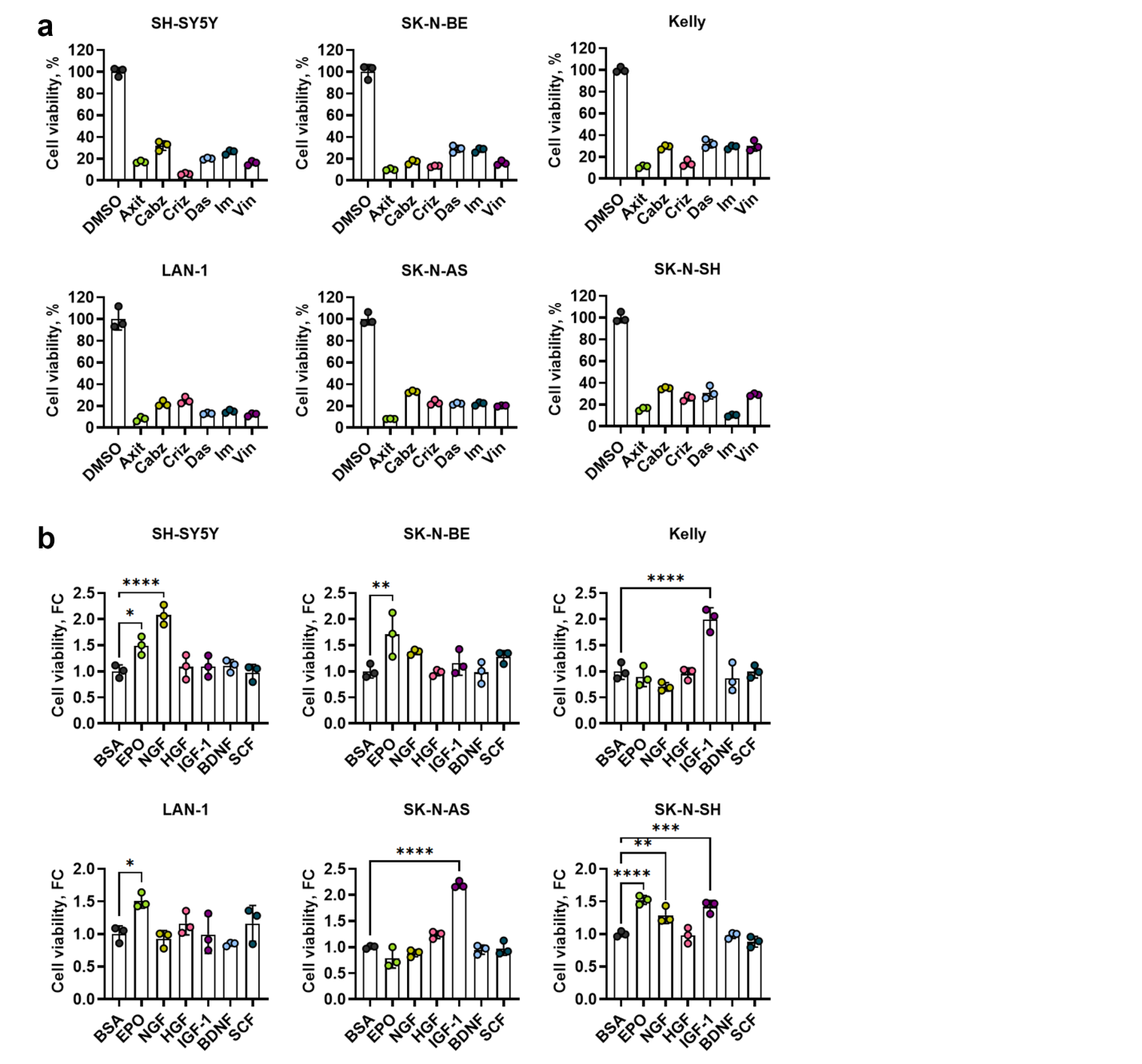


**Figure S7.** **Treatment of NB cells with vincristine in presence of growth factors. (a)** NB cells were treated as described on Figure 4a with axitinib (Axit), cabozantinib (Cabz), crizotinib (Criz), dasatinib (Das), imatinib (Im), and vincristine (Vin) with the addition of 0.1% BSA (as a mock-treatment used for further experiments). Cell viability in cells treated with DMSO was considered as 100%. **(b)** NB cells were treated with vincristine in presence of 100 ng/ml growth factors (EPO, NGF, HGF, IGF-1, BDNF, and SCF). Cells were treated as described on Figure 4a. Cell viability in cells treated with vincristine in combination with 0.1% BSA (mock treatment) was considered as 1. Mean values, individual data points where is possible, and SD are shown on graphs. *Mean and SD values are shown, Friedman test was used to determine statistically significant differences. *- p-value<0.05, **- <0.01, ***-<0.001, ****- < 0.0001*

**
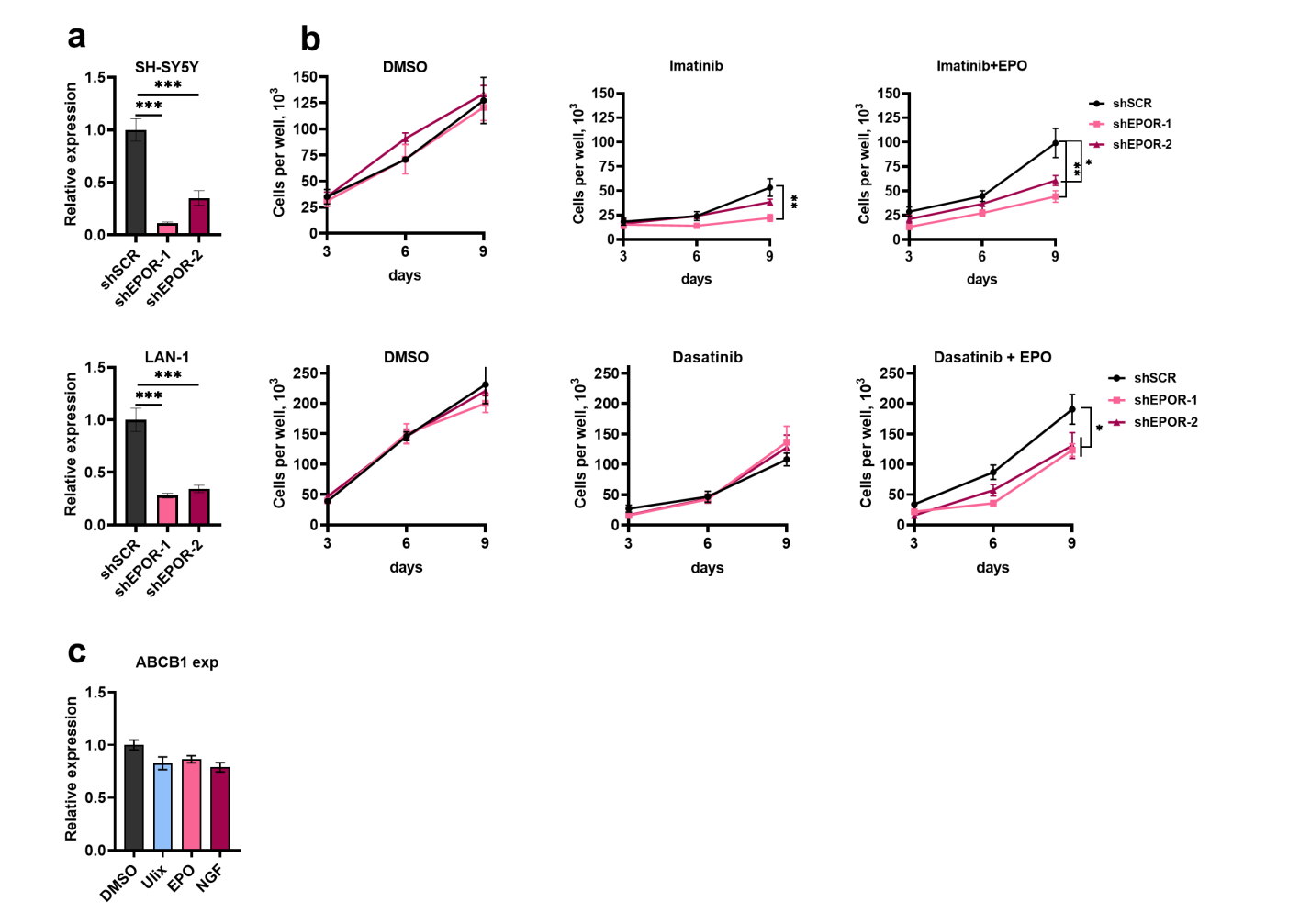
**

**Figure S8. EPOR knockdown does not affect NB cell proliferation. (a)** EPOR expression 72h after shRNA lentiviral transduction of SH-SY5Y and LAN-1 cells. shSCR- is a control shRNA with no targets among human mRNAs. **(b)** SH-SY5Y and LAN-1 cells survival after 3, 6 and 9 days after shRNA transduction and treatment with DMSO (mock treatment), imatinib, dasatinib, and their combination with 100 ng/ml EPO. **(c)** *ABCB1* gene expression in SH-SY5Y cells treated with 250 nM ulixertinib, and 100 ng/ml EPO or NGF.


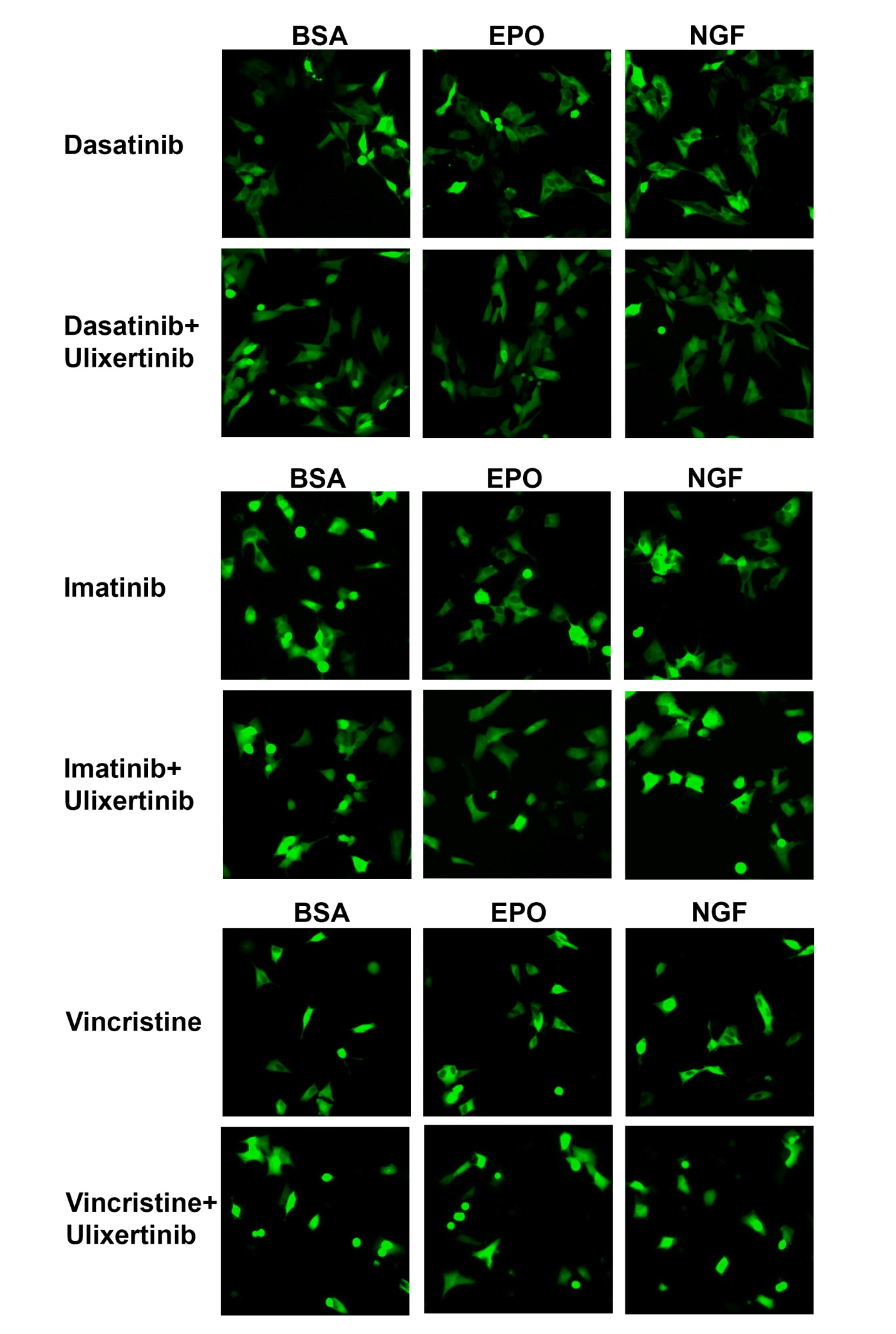


**Figure S9. Representative images of SH-SY5Y ERK-KTR.** Cells were exposed to imatinib (25 uM), dasatinib (25 nM), or vincristine (25 nM) for 72h, then serum starved and treated with DMSO or ulixertinib (250 nM) for 6 h, and after that EPO, NGF, or BSA (for control) were added. Images were taken 30 min after addition of growth factors. Translocation of fluorescent protein from nucleus to cytoplasm indicates ERK activation.

**
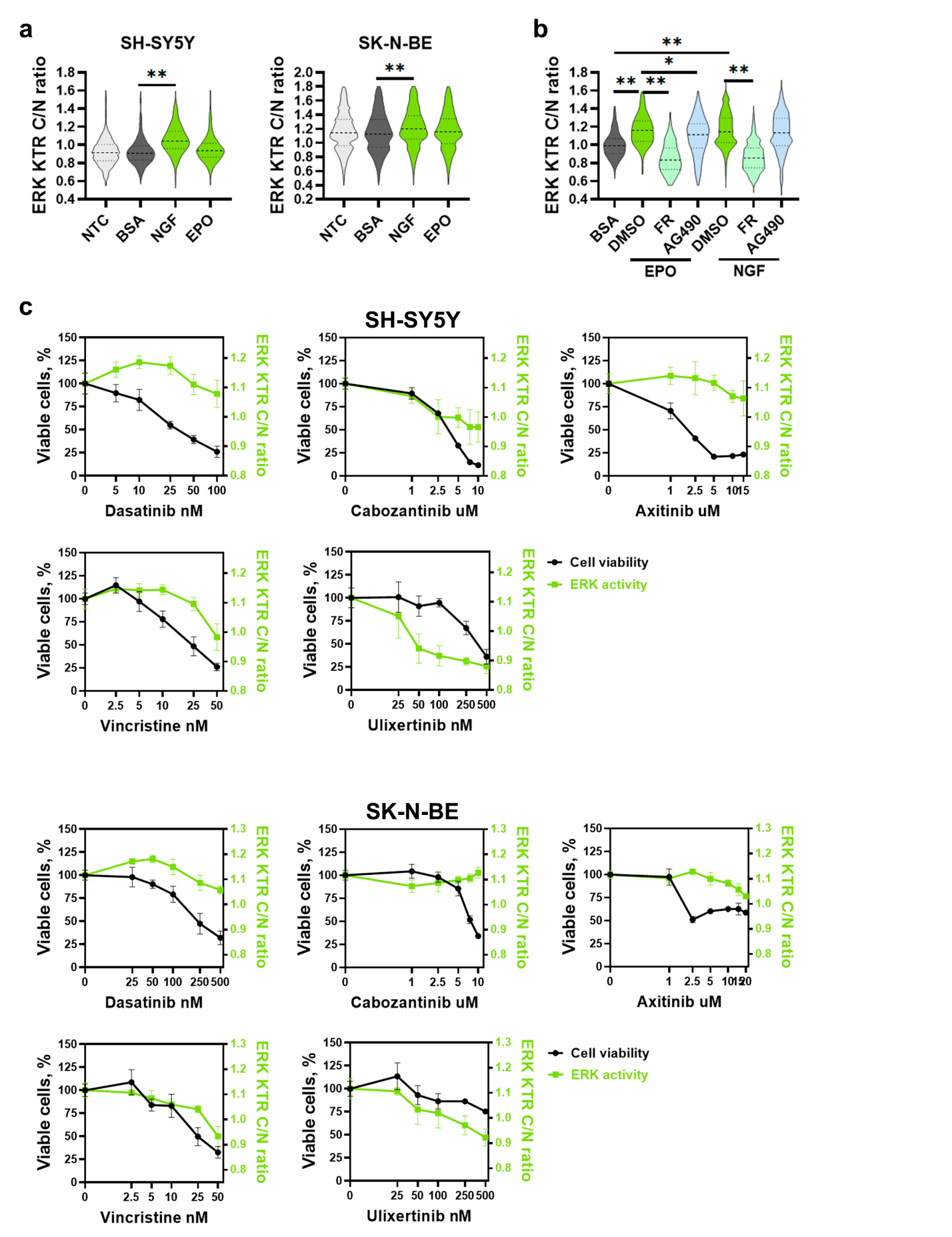
**

**Figure S10. EPO and NGF effect on ERK activity in NB cells. (a)** Violin plots show average distribution of ERK activity in cells treated for 30 min with recombinant growth factors (100 ng/ml each). Cells were serum starved for 6h prior to addition of growth factors. Cells were treated with 0.1% BSA as a mock treatment. NTC- non-treated control **(b)** SH-SY5Y cells were treated with imatinib for 72h and then the ability of EPO and NGF to activate ERK was measured as previously described, except cells were pretreated with 10 uM ERK1/2 inhibitor FR180204 or 7.5 uM JAK2 inhibitor AG490 prior to addition of growth factors. Violin plots show median values and 25th to 75th percentiles. **(c)** *ERK activity measured by ERK-KTR cytoplasm to nucleus ratio (C/N ratio) in SH-SY5Y and SK-N-BE 24h after treatment and cell viability measured 72h after treatment with dasatinib, cabozantinib, axitinib, vincristine, and ulixertinib.* *- p-value<0.05, **- p-value<0.01, ***- p-value<0.001 as calculated by Mann-Whitney U test.

**
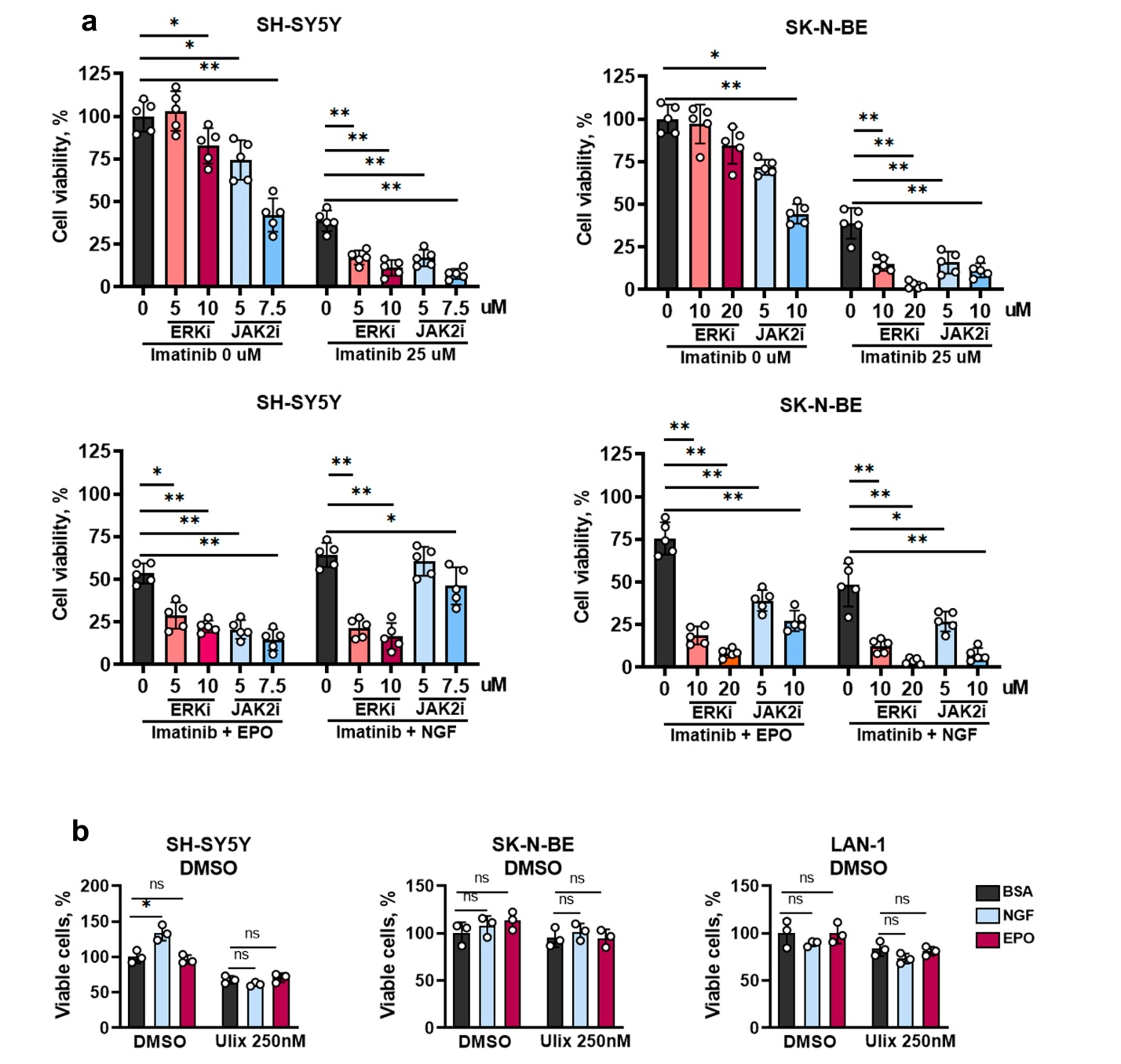
**

**Figure S11. Comparison of ERK and JAK2 inhibitors effect on EPO and NGF’s protective action. (a)** Cell viability of SH-SY5Y and SK-N-BE cells after treatment with imatinib in combination with FR180204 (ERKi) or AG490 (JAK2i) in the presence of EPO or NGF (100 ng/ml each). Cells were initially treated for 72h with imatinib, FR18024, AG490, EPO, and NGF simultaneously in different combinations. Then the growth medium was changed and cells were treated with fresh inhibitors and growth factors in the same manner for another 72h. **(b)** Cell viability of SH-SY5Y, SK-N-BE, and LAN-1 cells after treatment with ulixertinib in the presence of EPO or NGF (100 ng/ml each). Cells were treated as described previously for Figure 4. BSA (0.1%) was used as a carrier for reconstituted growth factors and the same amount of BSA was used as a control in experiments with EPO and NGF. DMSO was used as control for drug treatment. Mean values, individual data points, and SD are shown on graphs. *- p-value<0.05, **- p-value<0.01, ***- p-value<0.001 as calculated by Mann-Whitney U test.

**
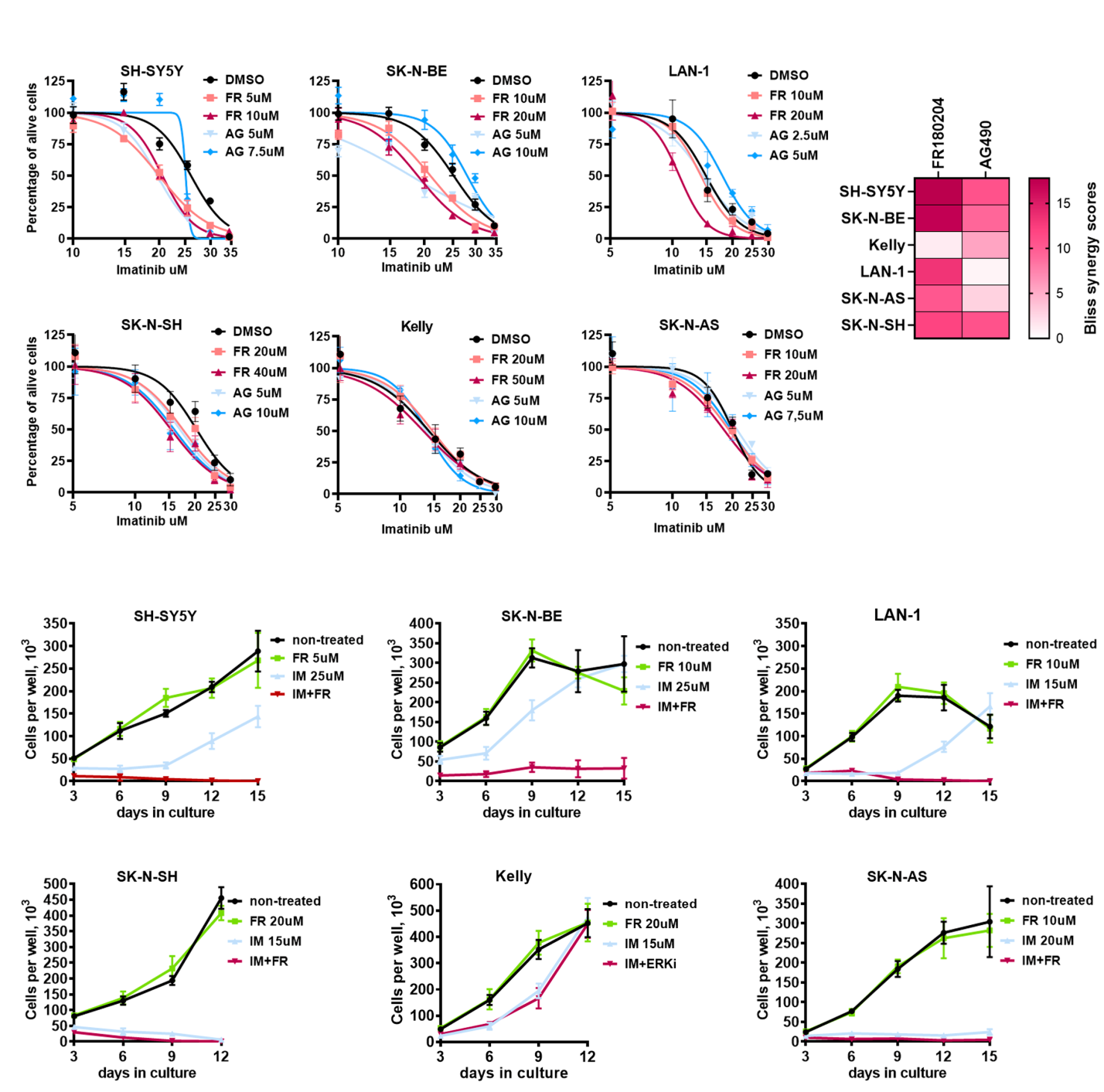
**

**Figure S12. NB cells survival in presence of ERK and JAK inhibitors. (a)** Cell viability of NB cells treated with imatinib in presence of ERK (FR180204) or JAK2 (AG490) inhibitors for 72h. For each drug combination number of cells not treated by imatinib was selected as 100%. Imatinib concentration is shown in logarithmic scale. DMSO was used for a control treatment. Heatmap shows synergy scores for each FR180204 and AG490 in combination with imatinib. **(b)** Cell growth curves for six NB cell lines treated with imatinib, ERK inhibitor FR180204 or in combination. Drugs were added simultaneously and growth medium was renewed every 72h with fresh drugs being added in the same concentrations. p-value<0.05, **- p-value<0.01, ***- p-value<0.001 as calculated by Mann-Whitney U test.

**
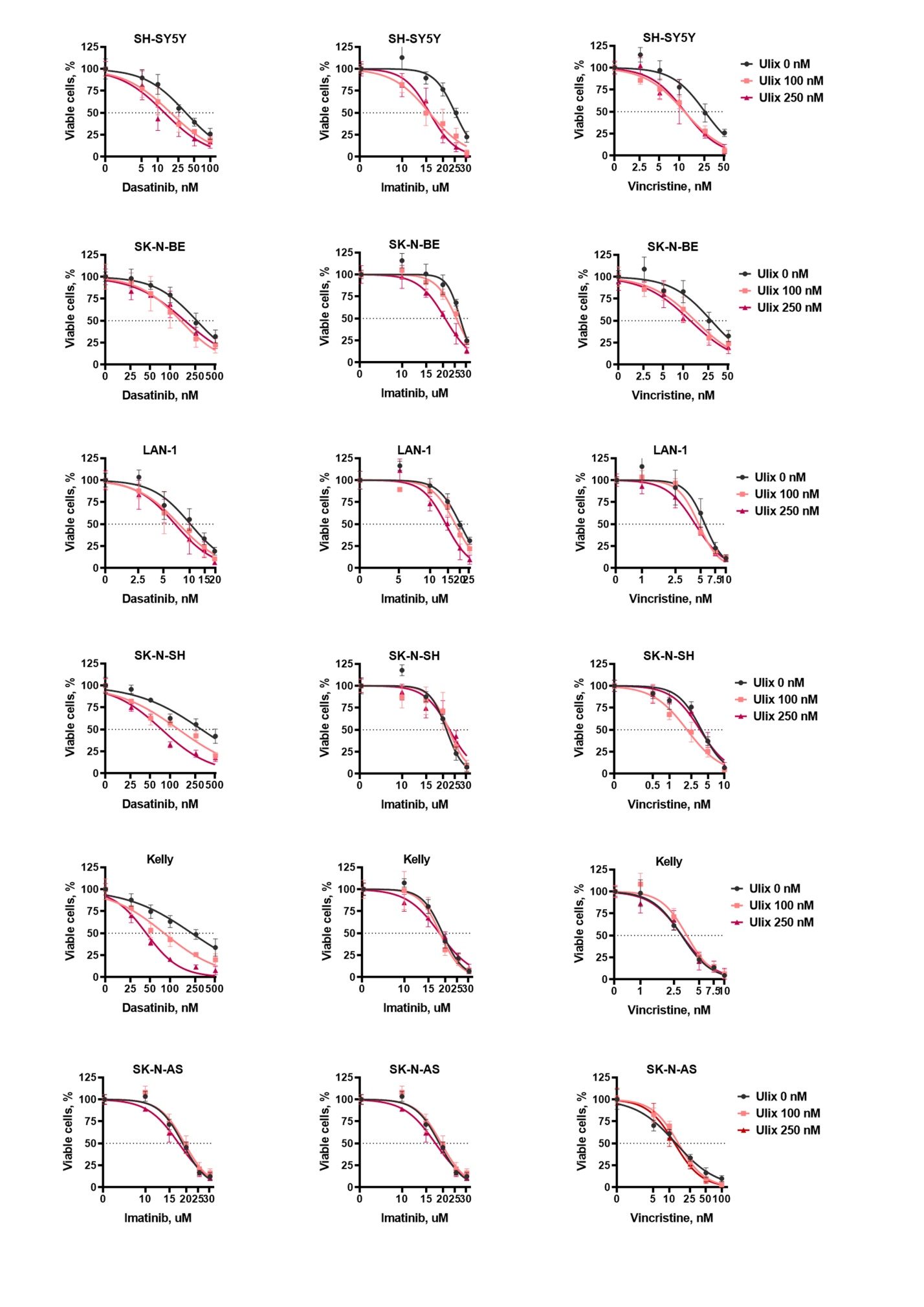
**

**Figure S13. NB cell viability when treated with imatinib, dasatinib, or vincristine in the presence of ulixertinib (Ulix) for 72h.** For each ulixertinib concentration used in combitnation with other drugs cell viability was normalized to number of cells treated only with ulixertinib. Drug concentrations are shown in logarithmic scale. DMSO was used for a control treatment.

**Table S1.** List of cell lines, drugs, growth factors, PCR primers, shRNAs, and datasets used in this study. Overall number of samples (n), GEO and R2 platform accession numbers, PMID numbers, and applications for respective publication are provided.

**Table S2. UMAP analysis of NB tumors.** List of 277 genes, encoding receptors, their ligands, and downstream kinases (Selected genes). UMAP and HDBSCAN clustering results, and normalized expression of 172 genes present in all datasets. DEGs for each cluster (when included or excluded from analysis) are provided on individual lists. Ratio- expression ratio in cluster vs. all other samples, p-adj- p-value after FDR correction.

**Table S3. Association of receptors with prognosis in NB datasets.** Association of gene high expression with overall survival and respective p-value after Bonferroni correction. Associations with overall survival were determined by Kaplan-Meier analysis using R2 platform.

**Table S4. Logistic regression model parameters.** Gene weights and intercept values for predicting outcomes of MYCN amplified and non-amplified NB tumors are provided on separate lists.

**Table S5. Clinical, gene expression, and pathway activation data for 60 NB patients.** NB- neuroblastoma, GNB- ganglioneuroblastoma, GN- ganglioneuroma, BM- bone marrow, DL- distant lymph nodes, Chem. blocks- number of NB2004 chemotherapy blocks received. Gene expression data for 60 NB tumors (Gene expression), and pathway activation level calculated for: all genes (PW all genes); genes differentially expressed in tumors with worse response to the therapy (PW response genes); genes differentially expressed in metastatic vs. localized tumors (PW metastasis genes). Differential analysis for pathways in metastatic vs. localized tumors, and in tumors with poor vs. good response to the therapy. P-value adjusted for multiple comparisons is provided.

**Table S6. List of GO gene sets identified by GSEA.** List of GO gene sets identified by GSEA for *EPOR*, *KIT*, *NTRK1*, *NTRK2*, *MET*, and *DDR2*. Each gene expression was used as a phenotype. Size- number of genes in each set, NES- normalized enrichment score.

**Table S7. Gene set prognostic scores.** Gene set prognostic scores for top 100 enriched gene ontology gene sets associated with *EPOR* expression, and 20 random gene sets. Values provided for three datasets: Versteeg (n=88), PrimaryNRC (n=283), and Kocak (n=476). Kaplan-Meier scan from R2: Genomics analysis and visualization platform ([http://r2.amc.nl](http://r2.amc.nl/)) was used to determine association of high gene expression with 5-year overall survival. “unfav”- number of genes in gene set that correlate with unfavorable prognosis; “fav”- number of genes in gene set that correlate with favorable prognosis; “not sign”- number of genes in gene set without statistically correlation with prognosis; “Mean”- mean prognostic score for three NB datasets; “p-value” and “q-value”- p-values for comparison of prognostic scores with scores for random gene sets before and after FDR correction.

**Table S8. Mutation analysis and drug selection.** Mutation frequencies (rates) for NB cell lines from CCLE dataset (CCLE_rate), patients from TARGET dataset (TARGET_rate), and ratio between frequencies in cell lines to patients (ratio) is provided on the Mutations tab. Mutations found in our panel of six NB cell lines are provided on “Mutations in cell lines” tab. Results of drugs found in DSigDB database and known drug targets are listed on “DSigDB drugs” tab.

**Table S9. Growth factor effects on NB cell survival.** Cell viability for each drug, growth factor and cell line is provided in three repeats, as well as mean viability changes. For each drug and cell line, cells treated with drug in combination with BSA was considered as 100%.

| **Cell line** | **Drug** | **DMSO** | **Ulix 100 nM** | **Ulix 250 nM** |
| --- | --- | --- | --- | --- |
| SH-SY5Y | Dasatinib, nM | 34,24 | 17,59 | 13,42 |
| SH-SY5Y | Imatinib, uM | 24,62 | 15,89 | 15,97 |
| SH-SY5Y | Vincristine, nM | 24,84 | 12,02 | 12,19 |
| SK-N-BE | Dasatinib, nM | 257,50 | 144,90 | 168,30 |
| SK-N-BE | Imatinib, uM | 26,78 | 25,13 | 20,75 |
| SK-N-BE | Vincristine, nM | 27,23 | 15,31 | 12,87 |
| SK-N-AS | Dasatinib, nM | 43,41 | 21,72 | 15,67 |
| SK-N-AS | Imatinib, uM | 18,83 | 19,48 | 17,61 |
| SK-N-AS | Vincristine, nM | 13,30 | 14,84 | 12,53 |
| SK-N-SH | Dasatinib, nM | 304,20 | 134,30 | 73,85 |
| SK-N-SH | Imatinib, uM | 21,24 | 22,09 | 22,39 |
| SK-N-SH | Vincristine, nM | 3,81 | 2,09 | 3,60 |
| LAN-1 | Dasatinib, nM | 10,26 | 7,49 | 6,74 |
| LAN-1 | Imatinib, uM | 20,07 | 17,91 | 14,43 |
| LAN-1 | Vincristine, nM | 5,61 | 4,74 | 4,30 |
| Kelly | Dasatinib, nM | 216,20 | 80,74 | 42,63 |
| Kelly | Imatinib, uM | 19,16 | 18,32 | 18,16 |
| Kelly | Vincristine, nM | 3,09 | 3,57 | 3,13 |

**Table S10.** **IC50 values for imatinib, dasatinib, and vincristine in combination with ulixertinib.**
